# Benchmarking high-dimensional mediation methods in epigenome-wide studies

**DOI:** 10.1093/bioadv/vbag331

**Published:** 2026-10-30

**Authors:** Wan-Yu Lin

**Affiliations:** Institute of Health Data Analytics and Statistics, College of Public Health, National Taiwan University, Taipei, 100025, Taiwan; Master of Public Health Program, College of Public Health, National Taiwan University, Taipei, 100025, Taiwan

## Abstract

**Motivation:**

High-dimensional mediation analysis is increasingly used in omics studies to investigate molecular mediators of exposure–outcome relationships. In epigenome-wide studies, the number of CpG sites often greatly exceeds the sample size, while strong correlations among sites challenge reliable mediator selection and effect estimation. We systematically benchmarked four high-dimensional mediation methods—HIMA, HIMA2, HDMA, and a strategy combining high-dimensional mediation testing with stepwise regression (HDMTS)—under simulation settings varying signal strength, correlation structure, and the mediator-to-sample ratio (*p*/*N*).

**Results:**

HIMA and HDMA consistently provided stringent false discovery proportion (FDP) control, whereas HIMA2 generally controlled FDP, with some inflation observed when the *p*/*N* ratio was high and mediator errors were independent. HDMTS showed greater sensitivity but exhibited reduced FDP control, particularly when mediator–outcome associations were weak and residual correlations among mediators were strong. Application to epigenome-wide DNA methylation data further demonstrated differences in mediator selection across methods and identified biologically plausible candidate mediators. Overall, our results highlight important trade-offs among false discovery control, sensitivity, and estimation accuracy and provide practical guidance for selecting high-dimensional mediation methods in epigenome-wide studies.

**Availability and implementation:**

The HDMTS code is available at https://github.com/WanYuLin/HDMTS, and the simulation code is available at https://github.com/WanYuLin/HDMTS/tree/main/Simulations.

## 1 Introduction

High-dimensional mediation analysis has emerged as an important statistical framework for investigating mechanisms linking exposures to complex outcomes in modern omics studies. In these settings, the number of candidate mediators often greatly exceeds the available sample size, and strong correlations among molecular features introduce substantial challenges for stable estimation and mediator selection. These issues are particularly pronounced in epigenome-wide mediation analysis (EWMA), where platforms such as the Illumina Infinium MethylationEPIC BeadChip (Illumina, Inc., San Diego, CA, USA) simultaneously quantify DNA methylation (DNAm) levels at more than 800 000 cytosine-phosphate-guanine (CpG) sites.

A mediation effect is typically characterized by an indirect pathway in which an exposure (*X*) influences an outcome (*Y*) through a mediator (*M*). Statistical inference is commonly based on two regression models, with α and β representing the exposure–mediator and mediator–outcome associations, respectively. The composite null hypothesis comprises three cases: (i) $H_{10}:\alpha \neq 0,\beta =0$; (ii) $H_{01}:\alpha =0,\;\beta \neq 0$; and (iii) $H_{00}:\alpha =0,\;\beta =0$. In high-dimensional settings, however, classical mediation procedures become unstable, motivating the development of specialized testing and estimation strategies.

Several methods have been proposed for EWMA by testing mediators sequentially, including Sobel’s test statistic (Sobel 1982), the MaxP test (also known as the joint significance test) (MacKinnon *et al.* 2002), the joint significance test under the composite null hypothesis (JT‐comp) (Huang 2019), the high‐dimensional mediation testing (HDMT) (Dai *et al.* 2022), the divide‐aggregate‐composite-null test (Liu *et al.* 2022), and a new variant of HDMT: Sobel‐comp (Du *et al*. 2023). A recent simulation study found that HDMT performed well across a range of evaluated scenarios (Du *et al.* 2023).

In addition to large-scale single-mediator hypothesis testing, multiple-mediator approaches are crucial because environmental factors may simultaneously influence DNAm at multiple CpGs. HIMA (Zhang *et al.* 2016), High-dimensional mediation analysis 2 (HIMA2) (Perera *et al.* 2022), and HDMA (Gao *et al.* 2019) are commonly used in EWMA. All three approaches use sure independence screening (SIS) (Fan and Lv 2008) to reduce the number of candidate CpGs to a manageable level. When assessing mediation effects, HIMA employs the minimax concave penalty (MCP) method, whereas HDMA and HIMA2 use a debiased least absolute shrinkage and selection operator (LASSO) procedure. All three methods use the joint significance principle to test mediation effects. HIMA2 further accounts for the composite null structure by modeling the null distribution as a mixture of the three component null cases, rather than treating mediation *P*-values under a single uniform null distribution. This mixture-null formulation explicitly accommodates the composite nature of the mediation null hypothesis and was introduced to improve false discovery control in high-dimensional mediation testing (Perera *et al.* 2022).

Despite their strong theoretical foundations and widespread application in EWMA, several studies have noted practical challenges associated with existing high-dimensional mediation approaches. HIMA has been reported to produce conservative inference under certain settings (Perera *et al.* 2022). HDMA and HIMA2 both adopt a debiased LASSO framework for inference, thereby alleviating regularization-induced bias. Nevertheless, these approaches rely on screening procedures and high-dimensional inference, whose performance may become challenging when signals are weak or dimensionality is extremely high (Fan and Lv 2008, Zhang and Zhang 2014, Dai *et al.* 2024).

In addition to these multiple-mediator approaches, a straightforward strategy is to first screen mediators using a single-mediator method, such as HDMT (Dai *et al.* 2022), and subsequently apply stepwise regression within a multiple-mediator model to select candidate mediators. However, to date, no head-to-head comparison has systematically evaluated the performance of this strategy relative to established high-dimensional mediation methods, including HIMA (Zhang *et al.* 2016), HIMA2 (Perera *et al.* 2022), and HDMA (Gao *et al.* 2019).

To address this gap, the present study systematically benchmarks four high-dimensional mediation methods under epigenome-wide settings through extensive simulations and a real-data application using DNA methylation data.

## 2 Methods

### 2.1 High‐dimensional mediation testing combined with stepwise regression

We consider a standard mediation framework for each CpG site *j*, in which the relationships among exposure, mediator, and outcome are modeled using two regression equations. Let *Y* be the trait, *X* be the exposure, *M_j_* be the DNAm level of CpG *j*, and ***C*** be the covariates, such as sex and age. A mediation analysis of CpG *j* (*j *= 1, …, *p*) incorporates a Model *M* and a Model *Y* as follows,


(1) $$M_{j}=\alpha_{0j}+\alpha_{j}X+\alpha_{C}^{T}C+\varepsilon_{M_{j}};\;(\mathrm{Model}\;M)\;$$

(2) $$Y=\beta_{0j}+\beta_{X}X+\beta_{j}M_{j}+\beta_{C}^{T}C+\varepsilon_{Y}.\;(\mathrm{Model}\;Y)\;$$

The coefficients α and β denote regression parameters in Models *M* and *Y*, respectively. $\varepsilon_{M_{j}}$ and $\varepsilon_{Y}$ are random error terms of Models *M* and *Y*, respectively. The mediation effect is tested by $H_{0}:\alpha_{j}\times \beta_{j}=0$ versus $H_{1}:\alpha_{j}\times \beta_{j}\neq 0$. The null hypothesis $H_{0}$ can be decomposed as three cases: (i) $H_{10}:\alpha_{j}\neq 0,\;\beta_{j}=0$; (ii) $H_{01}:\alpha_{j}=0,\;\beta_{j}\neq 0$; and (iii) $H_{00}:\alpha_{j}=0,\;\beta_{j}=0$. Let $\pi_{10}$, $\pi_{01}$, and $\pi_{00}$ be the proportions of the three classes of null hypothesis among all *p* CpGs.

The mediation effect for CpG site *j* is defined as $\alpha_{j}\times \beta_{j}$. Let the *P*-values of testing $\alpha_{j}=0$ and $\beta_{j}=0$ be $p_{\alpha j}$ and $p_{\beta j}$, respectively. Following the joint significance framework, the mediation effect is assessed using the maximum of the two *P*-values, defined as $p_{\max,j}=\max(p_{\alpha j},\;p_{\beta j})$. A large $p_{\max,j}$ indicates weak evidence against the null hypothesis. Assuming independence between $P_{\alpha j}$ and $P_{\beta j}$, the distribution of $P_{\max,j}$ over the three component null hypotheses (*H*_10_, *H*_01_, *H*_00_) forms a three-component mixture, whose cumulative distribution function (CDF) can be expressed as follows:


(3) $$\begin{array}{l} Pr(P_{\max,j}\leq p_{\max,j},H_{0})\\ =Pr(P_{\max,j}\leq p_{\max,j},H_{10})+Pr(P_{\max,j}\leq p_{\max,j},H_{01})+Pr(P_{\max,j}\leq p_{\max,j},H_{00})\\ \begin{array}{l} =Pr(P_{max,j}\leq p_{\max,j}|H_{10})\times Pr(H_{10})+Pr(P_{\max,j}\leq p_{\max,j}|H_{01})\times Pr(H_{01})\\ +Pr(P_{\max,j}\leq p_{\max,j}|H_{00})\times Pr(H_{00})\\ =\;Pr(P_{\alpha j}\leq p_{\max,j}|H_{10})\times Pr(P_{\beta j}\leq p_{\max,j}|H_{10})\times Pr(H_{10})\\ +Pr(P_{\alpha j}\leq p_{\max,j}|H_{01})\times Pr(P_{\beta j}\leq p_{\max,j}|H_{01})\times Pr(H_{01})\\ +Pr(P_{\alpha j}\leq p_{\max,j}|H_{00})\times Pr(P_{\beta j}\leq p_{\max,j}|H_{00})\times Pr(H_{00})\\ \begin{array}{l} =Pr(P_{\alpha j}\leq p_{\max,j}|H_{10})\times p_{\max,j}\times Pr(H_{10})+p_{\max,j}\times Pr(P_{\beta j}\leq p_{\max,j}|H_{01})\\ \times Pr(H_{01})+p_{\max,j}\times p_{\max,j}\times Pr(H_{00})\\ =\pi_{10}p_{\max,j}Pr(P_{\alpha j}\leq p_{\max,j}|H_{10})+\pi_{01}p_{\max,j}Pr(P_{\beta j}\leq p_{\max,j}|H_{01})+\pi_{00}p_{\max,j}^{2} \end{array} \end{array} \end{array}$$

where $Pr(P_{\alpha j}\leq p_{\max,j}|H_{10})$ and $Pr(P_{\beta j}\leq p_{\max,j}|H_{01})$ are the power of rejecting $\alpha_{j}=0$ under $H_{10}$ and the power of rejecting $\beta_{j}=0$ under $H_{01}$. When the sample size *N* is large enough, $Pr(P_{\alpha j}\leq p_{\max,j}|H_{10})$ and $Pr(P_{\beta j}\leq p_{\max,j}|H_{01})$ approaches 1, and $Pr(P_{\max,j}\leq p_{\max,j},H_{0})$ will approach $\pi_{10}p_{\max,j}+\pi_{01}p_{\max,j}+\pi_{00}p_{\max,j}^{2}=(\pi_{10}+\pi_{01})p_{\max,j}+\pi_{00}p_{\max,j}^{2}$. The asymptotic null distribution of $P_{\max,j}$ is therefore a mixture of uniform and Beta distributions: $\left(\pi_{10}+\pi_{01})U\mathrm{niform}(0,1)+\pi_{00}B\mathrm{eta}(2,1\right)$. With finite samples, $Pr(P_{\alpha j}\leq p_{\max,j}|H_{10})$ and $Pr(P_{\beta j}\leq p_{\max,j}|H_{01})$ can be estimated by the Grenander estimator (Grenander 1956). The three proportions ($\pi_{10}$, $\pi_{01}$, and $\pi_{00}$) can be estimated from the empirical distribution of $p_{\alpha j}$ and $p_{\beta j}$ (*j *= 1,…, *p*) using the multiple testing methods (Storey 2002).

Suppose some mediators were detected under the desired FDR (Benjamini and Hochberg 1995) control level, denoted $M_{k}\;(k\in I)$. The model for *Y* with multiple mediators is


(4) $$Y=\beta_{0}+\beta_{X}X+\sum_{k\in I}\beta_{k}M_{k}+\beta_{C}^{T}C+\varepsilon_{Y}.\;(\mathrm{Model}\;Y)$$

To select critical mediators from (4), stepwise regression based on the Akaike information criterion (AIC) was employed as a computationally efficient variable selection approach (Akaike 1974). If the variance inflation factor (VIF) of model (4) exceeds 5 (a commonly used indicator of multicollinearity), the least significant mediator is iteratively removed until VIF < 5. Finally, suppose |S| mediators ($M_{s},\;s\in S$; |S| is the number of elements in the set S) left in model (4), i.e.


(5) $$Y=\beta_{0}+\beta_{X}X+\sum_{s\in S}\beta_{s}M_{s}+\beta_{C}^{T}C+\varepsilon_{Y}.\;(\mathrm{Model}\;Y)$$

The estimated direct effect of *X* on *Y* is $\hat{\beta_{X}}$, and the mediation effect (or the indirect effect) is estimated as $\sum_{s\in S}\hat{\alpha_{s}}\hat{\beta_{s}}$, where the hat denotes the maximum likelihood estimate. The total effect of *X* on *Y* is the sum of direct and indirect effects, i.e. $\hat{\beta_{X}}+\sum_{s\in S}\hat{\alpha_{s}}\hat{\beta_{s}}$. When the total effect is nonzero, the mediation proportion belonging to mediator *s* is $\hat{\alpha_{s}}\hat{\beta_{s}}/\left(\hat{\beta_{X}}+\sum_{s\in S}\hat{\alpha_{s}}\hat{\beta_{s}}\right)$, where s∈S.

### 2.2 High-dimensional mediation analysis

High-dimensional mediation analysis (HIMA) (Zhang *et al.* 2016) consists of three steps. First, it uses the SIS method (Fan and Lv 2008) to select the top $d=\left\lceil 2N/\log(N)\right\rceil$ CpGs with the largest marginal effects on the outcome, $\left|\beta_{j}\right|$ (*j *= 1, …, *p*), where $\beta_{j}$ denotes the marginal effect of each CpG estimated from model (2), in which each mediator is included separately. $\left\lceil 2N/\log(N)\right\rceil$ is the smallest integer greater than or equal to 2N/log(N). This step reduces dimensionality from *p* ≫ *N* to a manageable scale while retaining CpGs that are most likely to be associated with the outcome. Second, variable selection is performed with the MCP (Zhang 2010). Suppose that the selected *d* CpGs form a subset I. HIMA (Zhang *et al.* 2016) estimates $\left\{\hat{\beta_{k}}:\;k\in I\right\}$ by minimizing the objective function:


(6) $$Q=\frac{1}{2N}\sum_{i=1}^{N}{\left(Y_{i}-\beta_{0}-\beta_{X}X_{i}-\sum_{k\in I}\beta_{k}M_{ik}-\beta_{C}^{T}C_{i}\right)}^{2}+\sum_{k\in I}p_{\lambda ,\delta }(\left|\beta_{k}\right|),$$

where the first term is the least squares loss and the second term is the MCP penalty applied to the mediator coefficients. The MCP penalty is defined as follows:


(7) $$p_{\lambda ,\delta }(\left|\beta_{k}\right|)=\{\begin{array}{l} \lambda |\beta_{k}|-\frac{\beta_{k}^{2}}{2\delta },\;if\;|\beta_{k}|\leq \lambda \delta \\ \frac{\lambda^{2}\delta }{2},\;if\;|\beta_{k}|>\lambda \delta \end{array}$$

The MCP penalty is imposed only on the mediator coefficients, $\beta_{k}$, k∈I. In (7), λ≥0 is the tuning parameter, and δ>1 controls the concavity. HIMA can be implemented using the “hima_classic” function in the HIMA R package. By default, δ is fixed at 3 [following the recommendation in the MCP paper (Zhang 2010)] and λ is selected via cross-validation. The shrinkage force of MCP on $\left|\beta_{k}\right|$ is the first derivative of MCP with respect to $\left|\beta_{k}\right|$, i.e.


(8) $$p_{\lambda ,\delta }\prime (\left|\beta_{k}\right|)=\{\begin{array}{l} \lambda -\frac{\left|\beta_{k}\right|}{\delta },\;if\;|\beta_{k}|\leq \lambda \delta \\ 0,\;if\;|\beta_{k}|>\lambda \delta \end{array}$$

As $\left|\beta_{k}\right|$ increases to λδ, the shrinkage force of MCP decreases to 0. When $|\beta_{k}|>\lambda \delta$, the shrinkage force vanishes. Compared with the LASSO, MCP reduces estimation bias for large coefficients by gradually diminishing the shrinkage force as $\left|\beta_{k}\right|$ increases.

Suppose that (6) selects a set S of mediators ($M_{k},\;k\in S$) with nonzero estimated coefficients $\hat{\beta_{k}}$. In practice, these selected mediators are refitted using the unpenalized linear model (5), which provides an empirical approximation to the variance under the MCP oracle property. The *P*-value for testing $H_{0}:\beta_{k}=0$ versus $H_{1}:\beta_{k}\neq 0$ is given by


(9) $$p_{\beta k}=2\{1-\Phi (\frac{\left|\hat{\beta_{k}}\right|}{\hat{\sigma_{\beta k}}})\}$$

where $\hat{\beta_{k}}$ and $\hat{\sigma_{\beta k}}$ are the ordinary least squares (OLS) estimate and its corresponding standard error, respectively; and Φ(·) denotes the CDF of the standard normal distribution. A normal approximation is employed based on the asymptotic normality of the estimator under the MCP framework. The Bonferroni-corrected *P*-value is $p_{Bc,\beta k}=\min(|S|\cdot p_{\beta k},1)$.

Similarly, the *P*-value for testing $H_{0}:\alpha_{k}=0$ versus $H_{1}:\alpha_{k}\neq 0$ in the exposure-mediator model is


(10) $$p_{\alpha k}=2\{1-\Phi (\frac{\left|\hat{\alpha_{k}}\right|}{\hat{\sigma_{\alpha k}}})\}$$

where $\hat{\alpha_{k}}$ and $\hat{\sigma_{\alpha k}}$ are the OLS estimate and its corresponding standard error, respectively. The Bonferroni-corrected *P*-value is $p_{Bc,\alpha k}=\min(|S|\cdot p_{\alpha k},1)$. The Bonferroni-corrected *P*-value for the joint significance test is $p_{Bc,k}=\max(p_{Bc,\alpha k},p_{Bc,\beta k})$. A mediator $M_{k}$ (k∈S) is declared significant if it is significant in both the exposure-mediator and mediator-outcome models. Specifically, $M_{k}$ (k∈S) is considered to have a statistically significant mediation effect if $p_{Bc,k}<\alpha$, where α is the nominal significance level (e.g. 0.05).

### 2.3 High-dimensional mediation analysis 2

While HIMA has been widely used for high-dimensional mediation analysis, its inference procedure assumes a simple (uniform) null distribution for *P*-values under the null hypothesis. However, this assumption may not hold under the composite null hypothesis (i.e. *H*_10_, *H*_01_, and *H*_00_) inherent in mediation analysis, where multiple configurations of null effects exist. To better account for this composite null structure, HIMA2 adopts a mixture null distribution for *P*-value calculation, thereby improving control of false positives.

There are three steps in HIMA2 (Perera *et al.* 2022). First, Perera *et al.* (2022) used the SIS method (Fan and Lv 2008) to select the top $d=\left\lceil 2N/\log(N)\right\rceil$ CpGs with the largest absolute mediation effects, $\left|\alpha_{j}\times \beta_{j}\right|$ [*j *= 1, …, *p*; where $\alpha_{j}$ and $\beta_{j}$ are marginal estimates obtained from (1) and (2), respectively]. This constitutes a key distinction between HIMA and HIMA2: HIMA ranks CpGs by the magnitude of $\left|\beta_{j}\right|$, whereas HIMA2 prioritizes CpGs based on the magnitude of the marginal mediation effect $\left|\alpha_{j}\times \beta_{j}\right|$. In Step 2, HIMA employs MCP, whereas HIMA2 adopts the debiased LASSO (Javanmard and Montanari 2014, Van de Geer *et al.* 2014, Zhang and Zhang 2014). Suppose that the *d* prioritized CpGs form a subset I. Conditional on the CpGs prioritized in Step 1, the LASSO estimator $\left\{\hat{\beta_{k}}:\;k\in I\right\}$ is obtained by minimizing the objective function:


(11) $$Q=\sum_{i=1}^{N}{\left(Y_{i}-\beta_{0}-\beta_{X}X_{i}-\sum_{k\in I}\beta_{k}M_{ik}-\beta_{C}^{T}C_{i}\right)}^{2}+\lambda \sum_{k\in I}\left|\beta_{k}\right|$$

where λ≥0 is the penalty parameter.

To facilitate statistical inference, HIMA2 further applies the debiased LASSO (Javanmard and Montanari 2014, Van de Geer *et al.* 2014, Zhang and Zhang 2014) to reduce the bias introduced by the L1 penalty. The debiased LASSO estimator for $\beta_{k}\;(k\in I)$ is calculated by


(12) $$\tilde{\beta_{k}}=\hat{\beta_{k}}+\frac{1}{N}w_{k}^{T}M^{T}(Y-M\hat{\beta })$$

where $\hat{\beta }$ denotes the LASSO estimator, ***M*** is the design matrix for the selected CpGs after adjusting for the exposure and covariates, and $w_{k}$ is a loading vector obtained via nodewise LASSO regression (Van de Geer *et al.* 2014). Unlike HIMA, HIMA2 does not refit an unpenalized linear model (5). Statistical inference is conducted directly using the debiased LASSO estimator, whose asymptotic normality under regularity conditions justifies statistical inference. Under regularity conditions, $\tilde{\beta_{k}}$ is asymptotically normally distributed (Van de Geer *et al.* 2014), allowing the construction of confidence intervals and hypothesis tests for individual CpGs.

In Step 3, as with HIMA, HIMA2 also performs joint significance testing. The *P*-value for the joint significance test is $p_{k}=\max(p_{\alpha k},p_{\beta k})$, where $p_{\alpha k}$ is obtained from (10) and $p_{\beta k}$ is calculated from


(13) $$p_{\beta k}=2\{1-\Phi (\frac{\left|\tilde{\beta_{k}}\right|}{\tilde{\sigma_{\beta k}}})\}$$

In (13), $\tilde{\beta_{k}}$ is the debiased LASSO estimate and $\tilde{\sigma_{\beta k}}$ is the corresponding standard error. In contrast to the Bonferroni correction used in HIMA, HIMA2 adopts an FDR-based approach to improve power while accounting for composite null hypotheses in high-dimensional mediation settings. FDR is controlled using the composite null framework implemented in the HDMT package (Dai *et al.* 2022), which estimates the proportions of partial null components ($\pi_{10}$, $\pi_{01}$, and $\pi_{00}$) based on the joint distribution of exposure-mediator and mediator-outcome *P*-values.

HDMA (Gao *et al.* 2019) employs SIS for mediator screening and debiased LASSO for high-dimensional inference. Unlike HIMA2, which uses an HDMT-based composite null framework that accounts for multiple partial null components, HDMA assumes a uniform null distribution of mediation *P*-values. The main analytical characteristics of the four methods are summarized in Table 1.

**Table 1:** Comparison of the main analytical characteristics of the four high-dimensional mediation methods.^a^

Analytical step	**HIMA (** Zhang et al. 2016 **)**	HDMA (Gao et al. 2019)	HIMA2 (Perera et al. 2022)	HDMTS
**Initial screening**	SIS	SIS	SIS	HDMT screening (Dai et al. 2022)
**Screening criterion**	|β|	|β|	|αβ|	Joint $p_{\alpha }\;\mathrm{and}\;p_{\beta }$
**High-dimensional inference**	Minimax concave penalty	Debiased LASSO	Debiased LASSO	Stepwise regression + VIF pruning
**Null modeling**	Uniform	Uniform	Mixture null	Mixture null
**Multiple testing correction**	Bonferroni (FWER control)	User-specified (e.g. FWER or FDR control)	FDR control	FDR control

a All four methods are based on the joint significance principle but differ in their screening strategies, high-dimensional inference procedures, null modeling, and multiple testing correction.

b FDR, false discovery rate; FWER, family-wise error rate; SIS, sure independence screening; VIF, variance inflation factor.

### 2.4 Simulation studies

Simulation studies were conducted to evaluate mediator selection performance, estimation accuracy, statistical power, and false discovery control under varying dimensionality and correlation structures. The simulation framework followed the design proposed in the HIMA2 study (Perera *et al.* 2022) to enable direct methodological comparison. The exposure *X* was generated from *N*(0, 2), a normal distribution with mean 0 and variance 2. Covariates $C={\left(C_{1},C_{2}\right)}^{T}$, where $C_{1}$ and $C_{2}$ were independently generated from *N*(0, 2). *p* CpGs were generated by


(14) $$M_{j}=\alpha_{j}X+0.3C_{1}+0.3C_{2}+\varepsilon_{j};\;(j=1,\ldots ,p)$$

The outcome *Y* was simulated by


(15) $$Y=0.5X+\sum_{j=1}^{p}\beta_{j}M_{j}+0.5C_{1}+0.5C_{2}+\varepsilon_{Y}$$

Using the setting from the HIMA2 paper (Perera *et al.* 2022), $\alpha_{1}=0.20$, $\alpha_{2}=0.25$, $\alpha_{3}=0.15$, $\alpha_{4}=0.30$, $\alpha_{5}=0.35$, $\alpha_{7}=0.10$, and $\alpha_{j}=0$ for all other *j*’s; $\beta_{1}=0.20$, $\beta_{2}=0.25$, $\beta_{3}=0.15$, $\beta_{4}=0.30$, $\beta_{5}=0.35$, $\beta_{6}=0.10$, and $\beta_{j}=0$ for all other *j*’s. Under this setting, five CpGs represented true mediators ($\alpha_{j}\beta_{j}\neq 0\;\mathrm{for}\;j=1,\;\ldots ,\;5$), while CpGs 6 and 7 corresponded to partial-null cases. The error term of Model M was $\varepsilon ={\left(\varepsilon_{1},\ldots ,\varepsilon_{p}\right)}^{T}$, generated from $N(0,\Sigma_{\varepsilon })$, where $\Sigma_{\varepsilon }={\left(\rho^{\left|j-j\prime \right|}\right)}_{p\times p}$ for *j*, *j*’ = 1, …, p. The error term of Model Y, $\varepsilon_{Y}$, was generated from the standard normal distribution.

A total of 16 scenarios were considered by considering three parameters: *P *= 1000 or 5000; *N *= 300 or 600; and ρ=0, 0.25, 0.50, or 0.75 (Perera *et al.* 2022). To further evaluate performance under weak mediation signals commonly observed in EWMA, additional scenarios were created by reducing effect sizes so that only one-half or one-third of $\alpha_{j}$ and $\beta_{j}$ were retained. In each replication, HDMT combined with stepwise regression was compared with HIMA (Zhang *et al.* 2016), HIMA2 (Perera *et al.* 2022), and HDMA (Gao *et al.* 2019).

To better reflect the local block structure of DNAm, we additionally considered a block-structured correlation scenario. CpGs were partitioned into blocks of 100 each. Within each block, correlations were modeled using a first-order autoregressive [AR(1)] structure, decreasing with increasing distance between CpGs. Specifically, within each block, the covariance matrix of the error term was defined as $\Sigma_{\varepsilon ,b}={\left(\rho^{\left|j-j\prime \right|}\right)}_{100\times 100}$ for *j*, *j′* = 1, …, 100, with *ρ* = 0, 0.25, 0.50, or 0.75. The covariance matrix for all *p* CpGs was then constructed as a block-diagonal matrix, with zero correlations between different blocks.

HIMA was implemented using the “hima_classic” function in the HIMA R package, with a Bonferroni-corrected *P*-value cutoff of 0.05 (Bonfcut = 0.05), which controls the family-wise error rate (FWER) as originally designed (Zhang *et al.* 2016). HIMA2 was implemented using the “hima_dblasso” function in the HIMA R package, with an FDR cutoff of 0.05 (FDRcut = 0.05), following its original specification for FDR control (Perera *et al.* 2022). The functions “hima_classic” and “hima_dblasso” were used to ensure consistency with the original procedures (Zhang *et al.* 2016; Perera et al. 2022). HDMA was performed using the R code downloaded from https://github.com/YuzhaoGao/High-dimensional-mediation-analysis-R/blob/master/HDMA.R, and the FDR cutoff was set at 0.05. These settings follow the default configurations for each method, allowing comparison within their intended error-control frameworks. Each simulation scenario was repeated 1000 times.

## 3 Results

### 3.1 Simulation results

Figure 1 shows the results using the $\alpha_{j}$ and $\beta_{j}$ values from Perera *et al*. (*j *= 1, …, *p*) (2022). In the simulation, five mediators were specified ($\alpha_{j}\beta_{j}\neq 0\;\mathrm{for}\;j=1,\;\ldots ,\;5$). Statistical power was defined as the proportion of true mediators correctly identified out of the five. For each simulation replication, we calculated the false discovery proportion (FDP) as the number of false discoveries divided by the total number of discoveries, setting FDP to zero when no discoveries were made. The mean FDP was calculated across all simulation replications to estimate the FDR. We also reported the proportion of replications with no discoveries for each method (Tables 1 and 2, available as supplementary data at *Bioinformatics Advances* online). Mean absolute error (MAE) was the average of the absolute difference between the estimated mediation effects ($\hat{\alpha_{j}}\hat{\beta_{j}}$) and the true mediation effects ($\alpha_{j}\beta_{j}$) for j=1, …, p. Computational time (in seconds) for each simulation replication was measured in R (version 4.3.1) on a Linux system with a 3.6 GHz processor and 32 GB of RAM.

**Figure 1:**
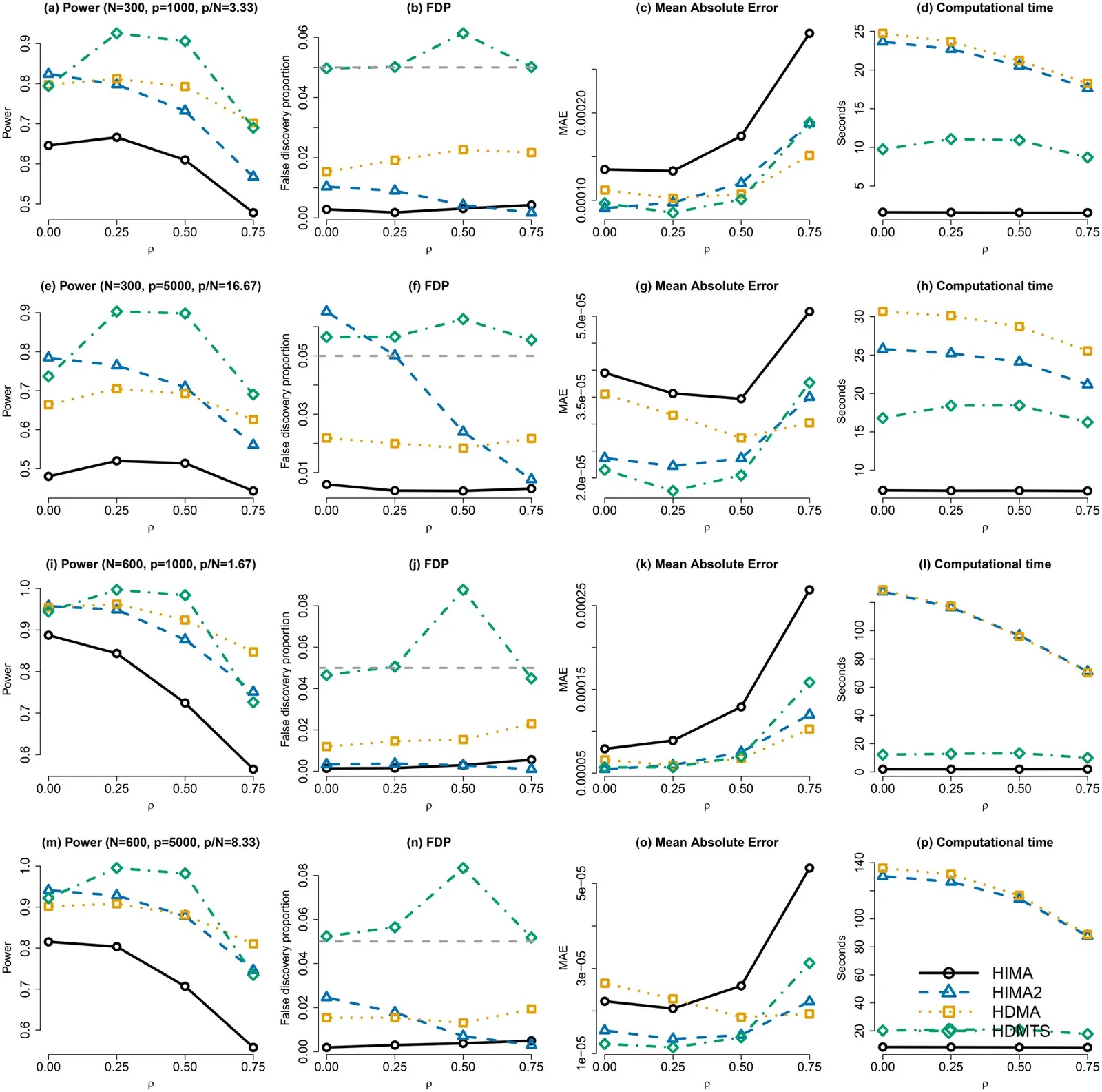
Simulation results under the simulation setting based on the $\alpha_{j}$ and $\beta_{j}$ values reported by Perera *et al*. (*j *= 1, …, *p*). Results are averaged over 1000 simulation replications. The columns (from left to right) show statistical power (panels a, e, i, and m), false discovery proportion (FDP; panels b, f, j, and n), mean absolute error (MAE) of the estimated mediation effect (panels c, g, k, and o), and computational time (seconds; panels d, h, l, and p). The rows (from top to bottom) correspond to the settings: *N* = 300, *p* = 1000; *N* = 300, *p* = 5000; *N* = 600, *p* = 1000; and *N* = 600, *p* = 5000. The *x*-axis denotes correlation levels among CpG sites (ρ=0, 0.25, 0.50, or 0.75). The gray horizontal line in the FDP panels indicates the nominal FDR level of 0.05.

**Table 2:** Summary of the strengths and limitations of the four high-dimensional mediation methods based on the simulation results.

Method	Strengths	Limitations
**HIMA (Zhang et al. 2016)**	Good FWER control; computationally efficient	Conservative with lower power; larger MAE (mean absolute error) for mediation effects
**HDMA (Gao et al. 2019)**	Generally higher power and lower MAE than HIMA	Longer computational time
**HIMA2 (Perera et al. 2022)**	Generally higher power and lower MAE than HIMA	FDP may slightly exceed the nominal level when the mediator-to-sample ratio (*p*/*N*) is high; power may decrease when exposure–mediator associations are weak, likely because the SIS screening statistic is based on |αβ|
**HDMTS**	Generally highest power	Generally higher FDP than the competing methods

HIMA2’s power generally decreased as the correlation among CpG levels (*ρ*) increased. In contrast, “HDMT combined with stepwise regression” (abbreviated as “HDMTS”) achieved the highest power under moderate correlation structures (*ρ* = 0.25 or 0.50). This increase in power was accompanied by elevated FDP, particularly at *ρ* = 0.50, suggesting that the higher sensitivity of HDMTS under moderate correlation came at the cost of less stringent false discovery control. Under strong correlation (*ρ* = 0.75), the power of HDMTS decreased substantially, although its FDP remained close to the nominal level. This reduction in power may partly reflect the difficulty of distinguishing true mediators from highly correlated CpGs during stepwise variable selection, as their individual mediator effects become increasingly difficult to disentangle.

Due to the conservative nature of the Bonferroni-corrected *P*-value, HIMA controlled FDP at a lower level than the other methods. HDMA also consistently controlled FDP under the nominal level (0.05). HIMA2 generally controlled FDP below the nominal level except when the *p*/*N* ratio was large and mediator errors were independent (ρ=0) (Fig. 1f). Under the baseline (strong-signal) setting, HDMTS exhibited a modest FDP inflation, especially when ρ = 0.50. The elevated FDP of HDMTS under moderate correlation (ρ = 0.50) may be attributed to partial correlation among mediators, which induces signal leakage from true mediators to correlated nonmediators. Given the relatively aggressive selection behavior of HDMTS, this leads to more false positives and, consequently, a higher FDP. This highlights the competing effects of correlation, which may facilitate signal aggregation but may also inflate FDP through signal leakage.

The relative ranking of MAE is stable across different correlation settings, with HIMA consistently yielding the largest error (column 3 of Fig. 1). This pattern suggests that the relative estimation efficiency of the competing methods is not strongly affected by the correlation structure among CpGs. MAE in Fig. 1 reflects overall estimation accuracy, whereas bias in Fig. 2 indicates systematic over- or underestimation. Figure 2e–h shows that HIMA and HDMA tend to underestimate the mediation effects, particularly in scenarios with larger *p*/*N* ratios.

**Figure 2:**
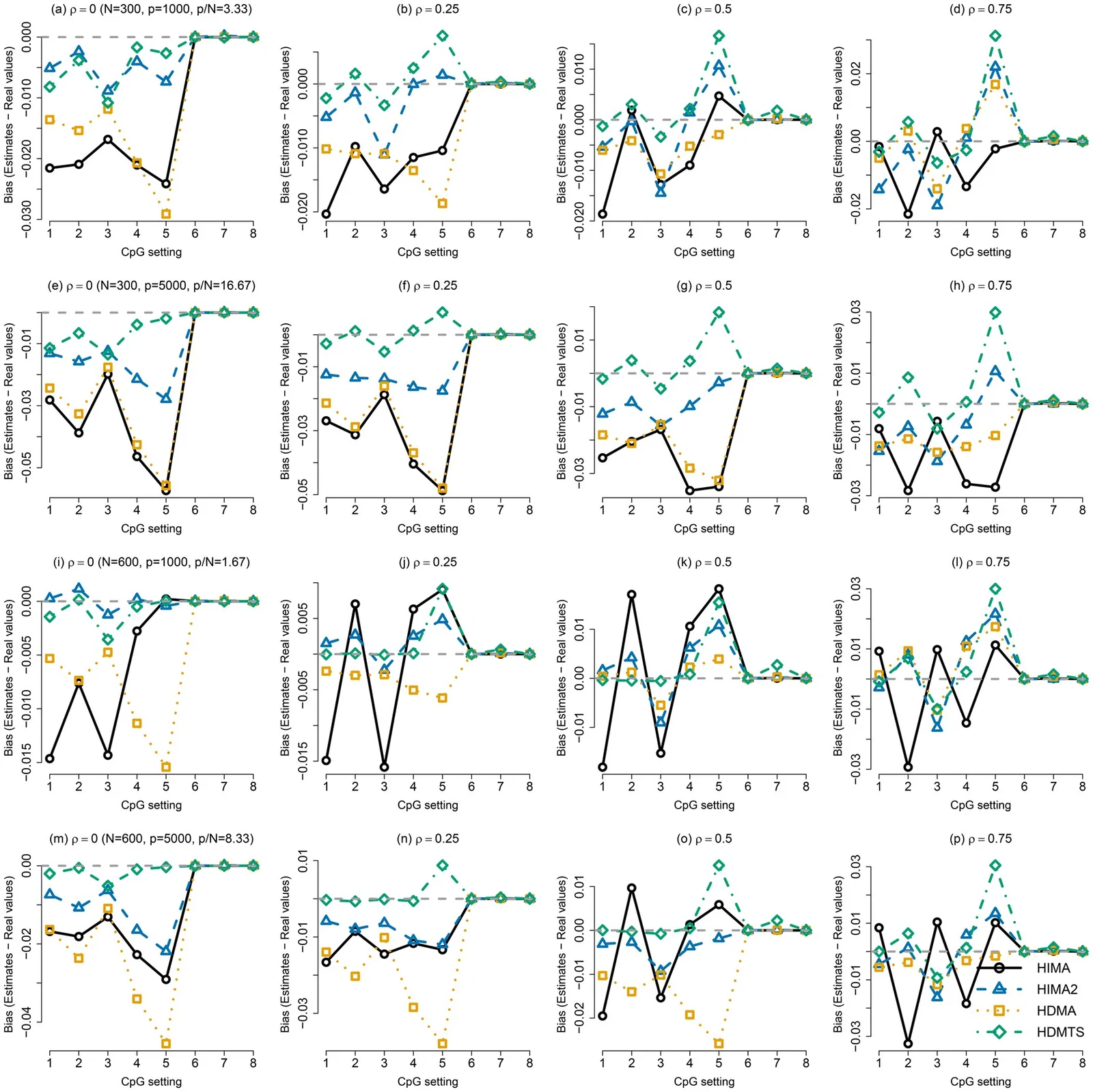
Bias of estimated mediation effects under the simulation setting based on the $\alpha_{j}$ and $\beta_{j}$ values from Perera *et al*. (*j *= 1, …, *p*). The rows (from top to bottom) correspond to the settings: *N* = 300, *p* = 1000; *N* = 300, *p* = 5000; *N* = 600, *p* = 1000; and *N* = 600, *p* = 5000. The columns (from left to right) represent correlation levels among CpG sites: *ρ* = 0 (panels a, e, i, and m), *ρ* = 0.25 (panels b, f, j, and n), *ρ* = 0.50 (panels c, g, k, and o), and *ρ* = 0.75 (panels d, h, l, and p). Bias was calculated as the average difference between estimated and true mediation effects over 1000 simulation replications. The *x*-axis denotes CpG settings corresponding to true nonzero effects: $\alpha_{1}=0.20$, $\alpha_{2}=0.25$, $\alpha_{3}=0.15$, $\alpha_{4}=0.30$, $\alpha_{5}=0.35$, $\alpha_{7}=0.10$, and $\alpha_{j}=0$ otherwise; $\beta_{1}=0.20$, $\beta_{2}=0.25$, $\beta_{3}=0.15$, $\beta_{4}=0.30$, $\beta_{5}=0.35$, $\beta_{6}=0.10$, and $\beta_{j}=0$ otherwise. CpG setting 8 represents the average bias across all CpG sites with *j* ≥ 8. The gray horizontal lines indicate zero bias.

Although HDMA and HIMA2 both employ debiased LASSO for estimation, the observed underestimation in HDMA may be related to the screening step rather than the estimation procedure itself. Specifically, in step 1 (the SIS step), HDMA screens CpGs based solely on the mediator–outcome association ($\left|\beta_{j}\right|$), whereas HIMA2 uses a criterion more directly related to mediation effects, namely the product $\left|\alpha_{j}\times \beta_{j}\right|$. Consequently, in high-dimensional settings (large *p*/*N*) (Fig. 2e–h), true mediators with relatively weak mediator–outcome associations but non-negligible mediation effects are more likely to be excluded by HDMA during screening. This screening-induced exclusion could contribute to the systematic downward bias observed in the estimated mediation effects.

In terms of computational time, HIMA was the fastest method, followed by HDMTS, whose runtime included 1000 bootstrap resamples. HDMA and HIMA2 required substantially more time than the other two methods (Fig. 1d, h, l, and p). Their runtimes increased markedly as the sample size increased from 300 to 600, whereas the increases were more modest for HIMA and HDMTS. The higher computational costs of HDMA and HIMA2 may reflect the additional computations involved in their debiased LASSO procedures.

Figures 3 and 4 show the results for half of the $\alpha_{j}$ values (*j *= 1, …, *p*). As $\alpha_{j}$ values decreased, HDMTS became the most powerful method while generally controlling FDP at the desired level. Figures 5 and 6 show the results for half of the $\beta_{j}$ values (*j *= 1, …, *p*). HDMTS achieved the highest power, at the cost of inflated FDP. Comparing Figs 3 and 5, FDP inflation for HDMTS is more pronounced when $\beta_{j}$ values are reduced. In addition, Figs 1–4, available as supplementary data at *Bioinformatics Advances* online, show simulation results when the $\alpha_{j}$ and $\beta_{j}$ values were reduced to one-third of their original values.

**Figure 3:**
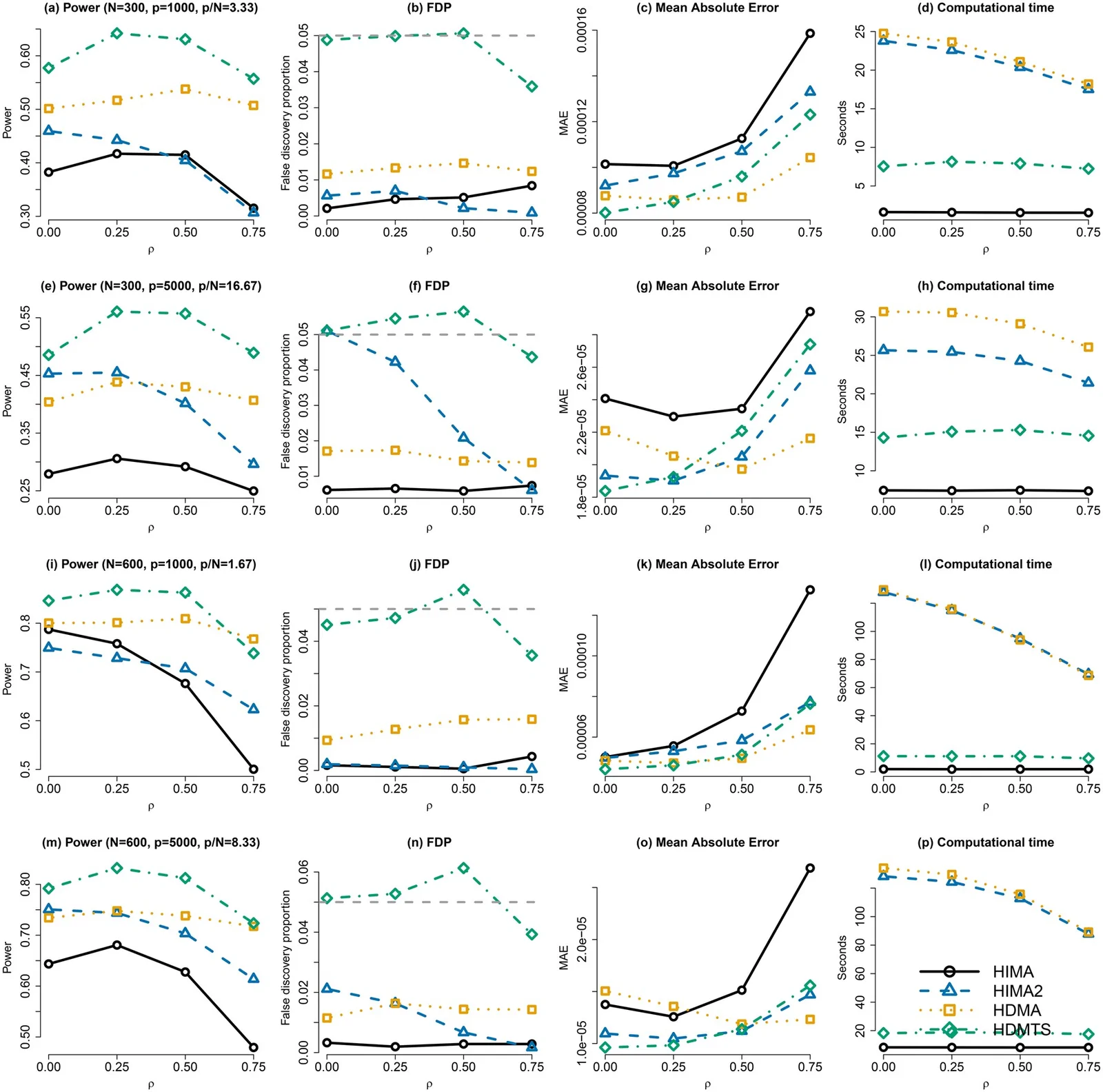
Simulation results under the simulation setting using half of the $\alpha_{j}$ values (*j *= 1, …, *p*). Results are averaged over 1000 simulation replications. The columns (from left to right) show statistical power (panels a, e, i, and m), false discovery proportion (FDP; panels b, f, j, and n), mean absolute error (MAE) of the estimated mediation effect (panels c, g, k, and o), and computational time (seconds; panels d, h, l, and p). The rows (from top to bottom) correspond to the settings: *N* = 300, *p* = 1000; *N* = 300, *p* = 5000; *N* = 600, *p* = 1000; and *N* = 600, *p* = 5000. The *x*-axis denotes correlation levels among CpG sites (ρ=0, 0.25, 0.50, or 0.75). The gray horizontal line in the FDP panels indicates the nominal FDR level of 0.05.

**Figure 4:**
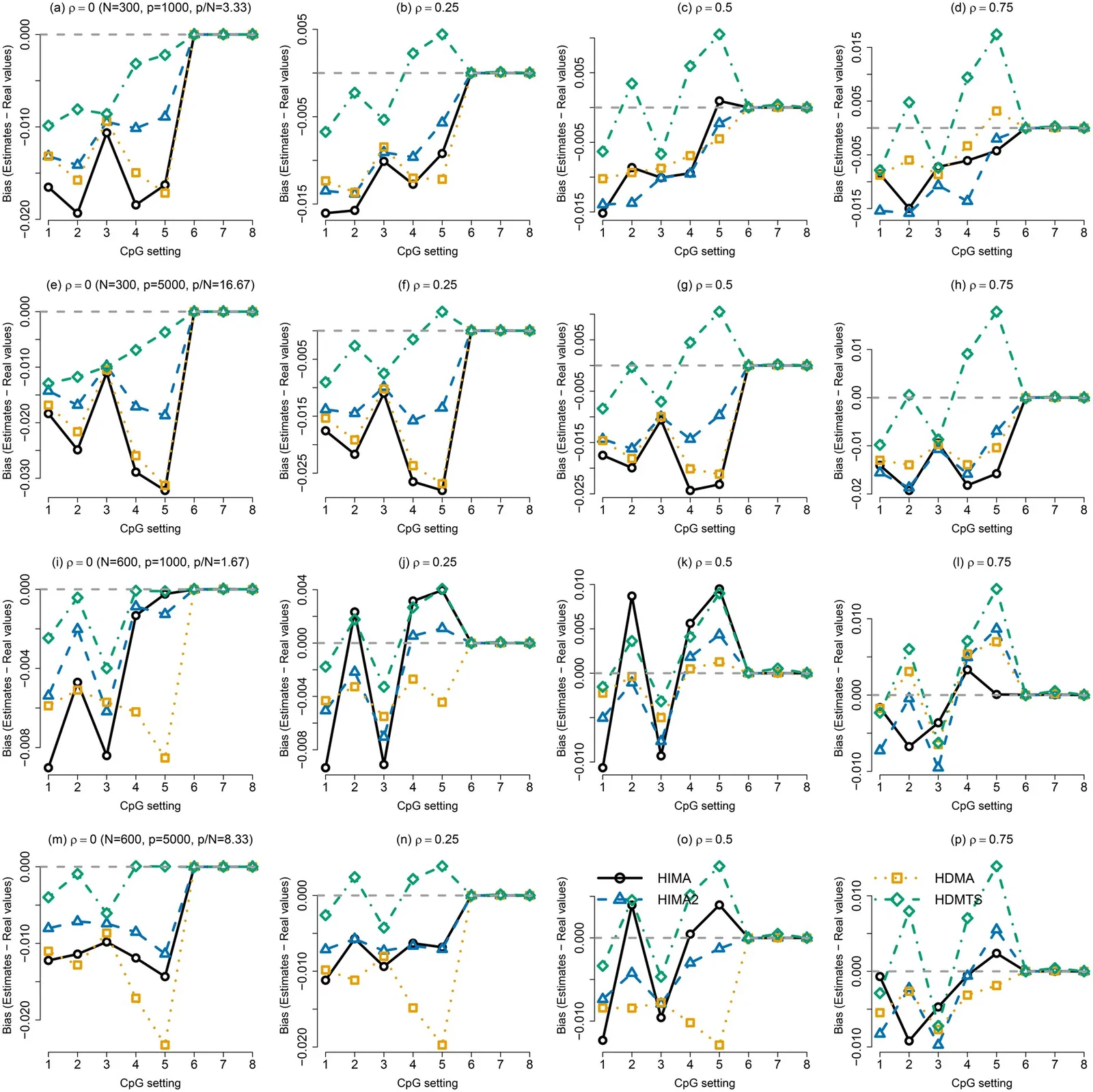
Bias of estimated mediation effects under the simulation setting using half of the $\alpha_{j}$ values (*j *= 1, …, *p*). The rows (from top to bottom) correspond to the settings: *N* = 300, *p* = 1000; *N* = 300, *p* = 5000; *N* = 600, *p* = 1000; and *N* = 600, *p* = 5000. The columns (from left to right) represent correlation levels among CpG sites (ρ=0, 0.25, 0.50, or 0.75). Bias was calculated as the average difference between estimated and true mediation effects over 1000 simulation replications. The *x*-axis denotes CpG settings corresponding to true nonzero effects: $\alpha_{1}=0.10$, $\alpha_{2}=0.125$, $\alpha_{3}=0.075$, $\alpha_{4}=0.15$, $\alpha_{5}=0.175$, $\alpha_{7}=0.05$, and $\alpha_{j}=0$ otherwise; $\beta_{1}=0.20$, $\beta_{2}=0.25$, $\beta_{3}=0.15$, $\beta_{4}=0.30$, $\beta_{5}=0.35$, $\beta_{6}=0.10$, and $\beta_{j}=0$ otherwise. CpG setting eight represents the average bias across all CpG sites with *j* ≥ 8. The gray horizontal lines indicate zero bias.

**Figure 5:**
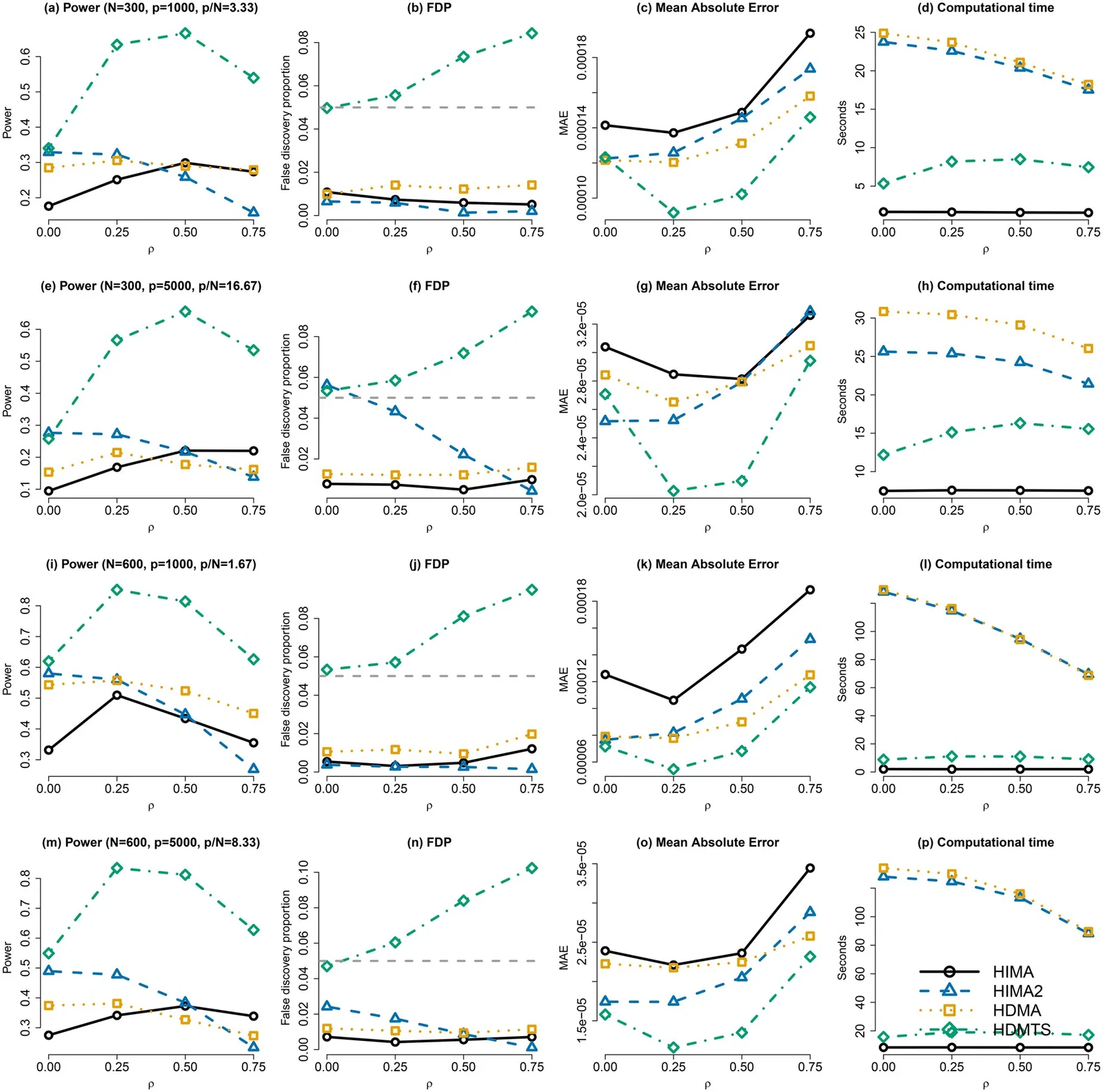
Simulation results under the simulation setting using half of the $\beta_{j}$ values (*j *= 1, …, *p*). Results are averaged over 1000 simulation replications. The columns (from left to right) show statistical power, false discovery proportion (FDP), mean absolute error (MAE) of the estimated mediation effect, and computational time (seconds). The rows (from top to bottom) correspond to the settings: *N* = 300, *p* = 1000; *N* = 300, *p* = 5000; *N* = 600, *p* = 1000; and *N* = 600, *p* = 5000. The *x*-axis denotes correlation levels among CpG sites (ρ=0, 0.25, 0.50, or 0.75). The gray horizontal line in the FDP panels indicates the nominal FDR level of 0.05.

**Figure 6:**
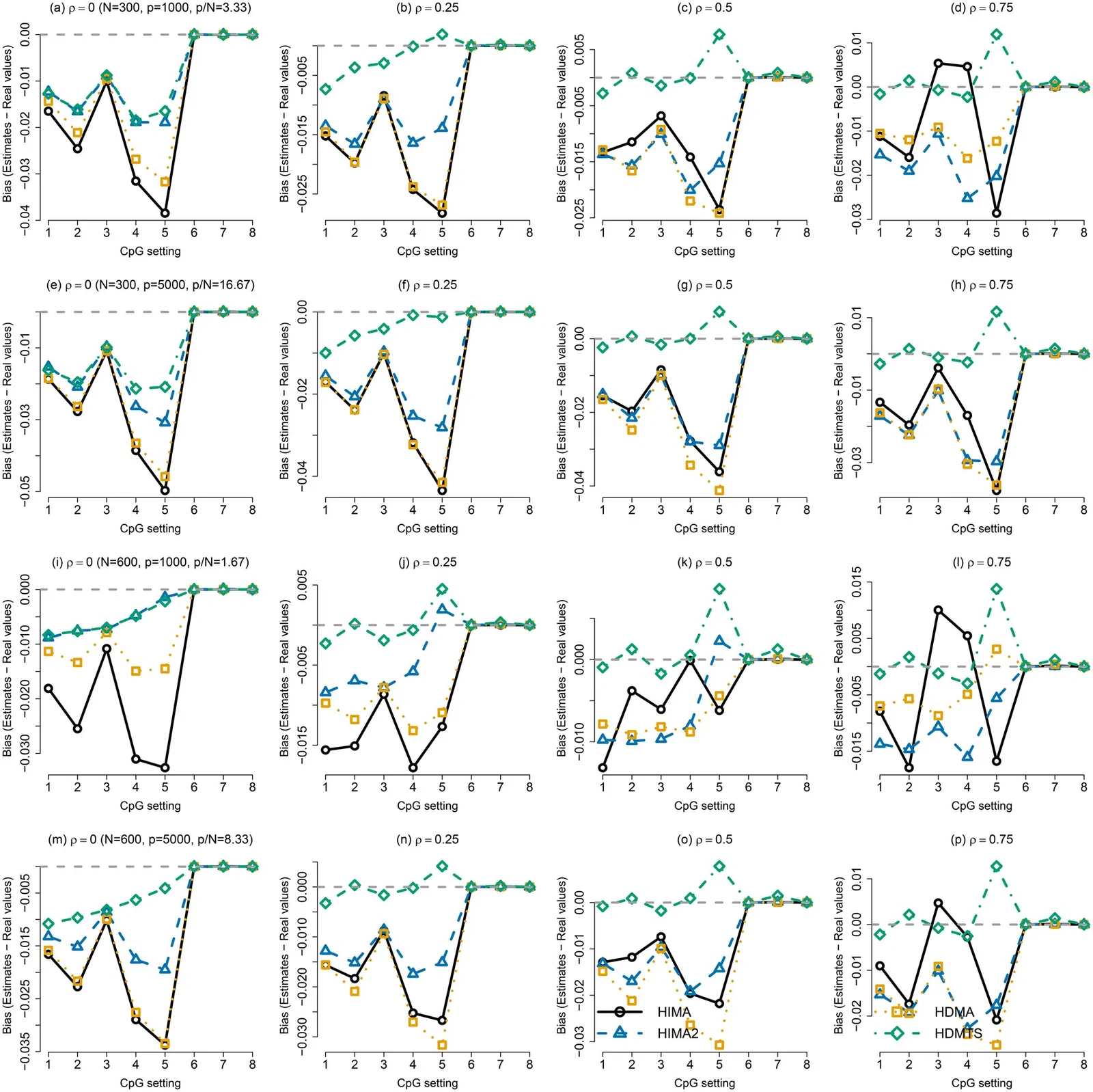
Bias of estimated mediation effects under the simulation setting using half of the $\beta_{j}$ values (*j *= 1, …, *p*). The rows (from top to bottom) correspond to the settings: *N* = 300, *p* = 1000; *N* = 300, *p* = 5000; *N* = 600, *p* = 1000; and *N* = 600, *p* = 5000. The columns (from left to right) represent correlation levels among CpG sites (ρ=0, 0.25, 0.50, or 0.75). Bias was calculated as the average difference between estimated and true mediation effects over 1000 simulation replications. The *x*-axis denotes CpG settings corresponding to true nonzero effects: $\alpha_{1}=0.20$, $\alpha_{2}=0.25$, $\alpha_{3}=0.15$, $\alpha_{4}=0.30$, $\alpha_{5}=0.35$, $\alpha_{7}=0.10$, and $\alpha_{j}=0$ otherwise; $\beta_{1}=0.10$, $\beta_{2}=0.125$, $\beta_{3}=0.075$, $\beta_{4}=0.15$, $\beta_{5}=0.175$, $\beta_{6}=0.05$, and $\beta_{j}=0$ otherwise. CpG setting 8 represents the average bias across all CpG sites with *j* ≥ 8. The gray horizontal lines indicate zero bias.

The FDP of HDMTS ranged from 0.05 to 0.10 when $\beta_{j}$ values were reduced by half (Fig. 5), and from 0.04 to 0.12 when $\beta_{j}$ values were reduced to one-third of their original values (Fig. 3, available as supplementary data at *Bioinformatics Advances* online). Reducing $\beta_{j}$ weakens the mediator–outcome association and, in HDMTS, can also hinder the stepwise selection of true mediators because stepwise regression is applied to the mediator–outcome model. This makes true mediators harder to retain, potentially reducing power and contributing to a higher FDP. In contrast, reducing $\alpha_{j}$ primarily weakens the exposure–mediator association but does not directly affect the stepwise selection step, resulting in a comparatively smaller impact on performance.

Overall, HDMTS showed greater sensitivity for mediator detection in most weak-signal settings, but its mean FDP generally exceeded the nominal level of 0.05. The FDP inflation was more pronounced under highly correlated mediator errors (*ρ* = 0.75) (Fig. 3b, f, j, and n, available as supplementary data at *Bioinformatics Advances* online). Correlation-induced signal leakage may explain this phenomenon.

HIMA, HIMA2, and HDMA generally exhibited more conservative selection behavior across the simulation scenarios. These three methods differ primarily in the screening statistic used in SIS. HIMA and HDMA screen mediators based on mediator–outcome association ($\left|\beta_{j}\right|$). In contrast, HIMA2 uses the product of the two associations ($\left|\alpha_{j}\times \beta_{j}\right|$), incorporating information from both the exposure–mediator and mediator–outcome relationships.

To better reflect local comethylation patterns, we additionally considered a block-structured correlation scenario. The simulation results (Figs 5–14, available as supplementary data at *Bioinformatics Advances* online) were largely consistent with those obtained under the original correlation structure, with no substantial changes in the overall comparative performance of the four methods.

Zero-discovery rates under both the original and block-structured correlation scenarios (Tables 1 and 2, available as supplementary data at *Bioinformatics Advances* online) were generally low under stronger-signal settings but increased substantially as the signals weakened, particularly when the sample size was small. The rates also differed considerably across methods in some weak-signal settings. In several such settings, HDMTS had lower zero-discovery rates than the other methods, consistent with its greater tendency to identify mediators.

Overall, the results highlight a fundamental trade-off between selection sensitivity and error control, which is jointly governed by signal strength and correlation structure. HDMTS favors sensitivity through more aggressive selection, whereas HIMA, HIMA2, and HDMA generally provide more stringent error control at the expense of power. Table 2 summarizes the strengths and limitations of the four high-dimensional mediation methods based on the simulation results.

### 3.2 Application to the Taiwan biobank data

The four methods were applied to the DNAm data obtained from the Taiwan Biobank (TWB). The TWB project was approved by the Ethics and Governance Council of the Taiwan Biobank and by the Institutional Review Board on Biomedical Science Research (IRB-BM) at Academia Sinica. Additionally, this study received approval from the Research Ethics Committee of the National Taiwan University Hospital (NTUH-REC no. 201805050RINB).

Quality control and normalization of DNAm data have been described previously (Lin 2026). Briefly, normalization was performed using the “noob” method (normal-exponential convolution using out-of-band probes) (Triche *et al.* 2013), as implemented in the “minfi” R package (Fortin *et al.* 2017), which reduces within- and between-platform technical artifacts while improving sensitivity.

Based on county-level population sizes and the male-to-female ratio in Taiwan, TWB randomly selected 2474 participants for DNAm quantification. After excluding 102 individuals diagnosed with diabetes (to avoid possible influences from medication) and six individuals with a mismatch between self-reported sex and DNAm-predicted sex, the final analytical sample included 2366 individuals (1172 men and 1194 women). Approximately 860 000 CpG sites were measured using the Illumina Infinium MethylationEPIC BeadChip (Illumina, Inc., San Diego, CA, USA).

HIMA was implemented using the “hima_classic” function in the HIMA R package, with a Bonferroni-corrected *P*-value cutoff of 0.05. HIMA2 was implemented using the “hima_dblasso” function in the HIMA R package, with an FDR cutoff of 0.05. HDMA was performed using the R code downloaded from https://github.com/YuzhaoGao/High-dimensional-mediation-analysis-R/blob/master/HDMA.R, and the FDR cutoff was set at 0.05. For HDMTS, 73 CpGs with HDMT FDR < 0.05 were subsequently entered into the stepwise selection procedure described in section 2.1.

Table 3 summarizes the CpGs identified as mediators of the association between body mass index (BMI) and fasting glucose. The HDMTS *P*-values reported in Table 3 should be interpreted descriptively, as they do not account for uncertainty introduced by the preceding data-driven variable-selection step. We treated BMI as the exposure and fasting glucose as the outcome based on prior evidence linking adiposity to glycemic traits, together with epigenome-wide association studies supporting associations among adiposity, DNAm, and glycemic outcomes (Hu *et al.* 2001, Chambers *et al.* 2015, Wahl *et al.* 2017). All four methods were adjusted for sex, age, smoking status, alcohol consumption status, regular physical exercise, educational attainment, and 11 immune-cell fractions estimated by the EpiDISH package (Luo *et al.* 2023): basophils, memory B-cells, naïve B-cells, memory CD4 + T-cells, naïve CD4 + T-cells, memory CD8 + T-cells, naïve CD8 + T-cells, eosinophils, monocytes, natural killer, and regulatory T-cells. Neutrophils were not adjusted due to multicollinearity.

**Table 3:** CpG mediators linking body mass index to fasting glucose (in mg/dL).

Method^a^	CpG site	eQTM Atlas^b^	Chr.	Gene	$\hat{\alpha_{j}}$	$\hat{\beta_{j}}$	**Mediation effect (** $\hat{\alpha_{j}}\hat{\beta_{j}}$ **)**	*p_max_* ^c^	Computational time
**HIMA**	cg19693031	V	*1*	*TXNIP*	−0.001467	−45.8553	0.0673	4.3451E − 7	1.08 hours
	cg04816311	V	*7*	*C7orf50*	0.001484	21.9027	0.0325	0.0006	
	cg21291385	V	*21*	*MRPS6*	−0.000531	−44.9436	0.0239	0.0004	
**HDMA**	cg19693031	V	*1*	*TXNIP*	−0.001467	−47.8596	0.0702	1.0869E − 7	2.09 hours
	cg15328937		2	*LOC101929452*	−0.000827	−54.9579	0.0455	7.0895E − 6	
	cg16958707		3	*FNDC3B*	−0.000390	−35.5818	0.0139	1.8353E − 3	
	cg14103229		3	*—*	−0.000987	−30.6208	0.0302	1.2966E − 3	
	cg00574958	V	11	*CPT1A*	−0.001383	−78.6220	0.1087	2.2742E − 4	
	cg05325763		11	*CPT1A*	−0.001747	−47.6156	0.0832	2.0912E − 3	
	cg21291385	V	21	*MRPS6*	−0.000531	−48.0940	0.0255	9.3326E − 4	
**HIMA2**	cg19693031	V	*1*	*TXNIP*	−0.001467	−60.1199	0.0882	6.8184E − 9	1.32 hours
**HDMTS**	cg19693031	V	*1*	*TXNIP*	−0.001467	−88.3578	0.1296	7.7363E − 9^d^	1.63 hours
	cg04816311	V	*7*	*C7orf50*	0.001484	43.9138	0.0652	2.6252E − 8^d^	

a All four methods were adjusted for sex, age, smoking status, alcohol consumption status, regular physical exercise, educational attainment, and 11 immune-cell fractions estimated by the EpiDISH package (Luo *et al.* 2023): basophils, memory B-cells, naïve B-cells, memory CD4 + T-cells, naïve CD4 + T-cells, memory CD8 + T-cells, naïve CD8 + T-cells, eosinophils, monocytes, natural killer, and regulatory T-cells. Neutrophils were not adjusted due to multicollinearity.

b “V” indicates that the CpG shows a significant association with gene expression according to the eQTM Atlas database (https://shiny.crc.pitt.edu/eqtm_browser/).

c *p*_max_ is the maximum of $p_{\alpha }$ and $p_{\beta }$.

d As HDMTS incorporates a data-driven variable-selection step before significance testing, the reported *P*-values do not account for model-selection uncertainty and should therefore be interpreted descriptively rather than as calibrated inferential quantities.

The numbers of CpGs identified by the four methods were HDMA (7), HIMA (3), HDMTS (2), and HIMA2 (1). Cg19693031 (*TXNIP*) was consistently identified as a significant mediator across all methods. HIMA and HDMTS additionally identified cg04816311 (*C7orf50*), while HIMA and HDMA detected cg21291385 (*MRPS6*). According to the eQTM Atlas database, these CpGs have been reported as expression quantitative trait methylation (eQTM) sites for genes including *TXNIP*, *GPR146*, and *MRPS6*.

*TXNIP* has been widely implicated in glucose metabolism and insulin signaling (Alhawiti *et al.* 2017). Hypomethylation at cg19693031 is associated with higher fasting glucose and increased diabetes risk (Li *et al.* 2022, Tsai *et al.* 2022). In line with previous studies (Li *et al.* 2022, Tsai *et al.* 2022), the estimated mediator–outcome association between cg19693031 and fasting glucose was negative, with estimates of -45.8553 (HIMA), -47.8596 (HDMA), -60.1199 (HIMA2), and -88.3578 (HDMTS), respectively. The smaller-magnitude estimates from HIMA, HDMA, and HIMA2 relative to HDMTS were qualitatively consistent with the estimation patterns observed under the high *p*/*N* simulation setting (Fig. 2e–h). However, without ground truth, it is not possible to determine whether the larger estimate from HDMTS reflects reduced conservativeness or overestimation. Cg04816311, identified by HIMA and HDMTS, has also been reported in multiple studies as a diabetes-associated CpG (Wang *et al.* 2021, Hong *et al.* 2022). By contrast, cg21291385, identified by HIMA and HDMA, has limited evidence linking it to glucose or diabetes. Its relevance to glucose metabolism remains unclear.

In addition to cg19693031 (*TXNIP*) and cg21291385 (*MRPS6*), HDMA solely identified five CpG sites: cg00574958 (*CPT1A*), cg05325763 (*CPT1A*), cg14103229, cg16958707 (*FNDC3B*), and cg15328937 (*LOC101929452*). Among these five CpGs, cg00574958 (*CPT1A*) has been consistently reported to be strongly associated with metabolic phenotypes such as triglycerides and glucose (Lai *et al.* 2020), whereas the remaining CpGs have comparatively less evidence linking them to diabetes-related traits. Overall, the overlap with previously reported CpGs supports concordance with the existing literature, although the real-data analysis was not designed as a formal replication study.

## 4 Discussion

While single-mediator models consider only one CpG site as a mediator, epigenetic studies suggest that multiple CpG sites often jointly mediate the effects of exposure on phenotypic outcomes (Opsasnick *et al.* 2024). In high-dimensional mediation analysis, HIMA and HDMA consistently controlled the FDP at relatively low levels. HIMA2 generally maintained FDP control, although some inflation was observed when the *p*/*N* ratio was high and mediator errors were independent. In contrast, HDMTS may exhibit reduced FDP control when mediator–outcome associations are weak. Together, these findings highlight the challenges of controlling false discoveries in high-dimensional settings, particularly when mediator–outcome associations are weak and mediators are correlated.

Simulation results indicated that HDMA and HIMA2 required substantially more computational time than HIMA and HDMTS, likely due to the computational burden of the debiased LASSO procedure. In the real data analysis, all four methods completed within 2.1 hours, although HDMA took the longest runtime.

Overall, all four methods can analyze large-scale EWMA datasets within a practical computational time. Their relative performance suggests that method selection should depend on the underlying signal structure and research objective. HIMA, HIMA2, and HDMA generally provide more stringent error control at the expense of statistical power, whereas HDMTS prioritizes sensitivity through more aggressive selection. Thus, when strict control of false discoveries is required, the more conservative methods may be preferred, whereas HDMTS may serve as a complementary approach for identifying additional candidate mediators, particularly when mediator–outcome associations are not too weak.

This study has several limitations. First, although we evaluated the methods under multiple simulation settings, the simulation framework cannot fully capture all characteristics of real DNAm data. In particular, simulations based directly on real DNAm data could better preserve important features of EPIC array data, such as probe-specific variability, heterogeneous noise, and more complex correlation structures. However, developing such a semi-synthetic simulation framework was beyond the scope of the present study. Second, because HDMTS incorporates a data-driven variable-selection step before significance testing, the reported *P*-values do not account for model-selection uncertainty. Therefore, statistical inference based on these *P*-values should be interpreted with appropriate caution. Our simulation evaluates the complete HDMTS procedure, including the model-selection step; thus, the reported empirical FDP reflects the observed operating characteristics of the procedure under the simulated settings. Future work could investigate postselection inference or other inference procedures that explicitly account for selection uncertainty, thereby providing more reliable statistical inference.

## 5 Conclusion

This study provides a comprehensive comparison of four high-dimensional mediation analysis methods for EWMA. The results demonstrate that methods emphasizing strict error control, such as HIMA, HIMA2, and HDMA, effectively limit false discoveries but may sacrifice statistical power. In contrast, HDMTS offers improved sensitivity by identifying a broader set of candidate mediators, while maintaining acceptable FDP control when mediator–outcome associations are not too weak.

These findings suggest that the choice of method should be guided by the primary research objective and the underlying signal structure. When minimizing false positives is critical, conservative approaches may be preferred, whereas HDMTS may serve as a useful complementary tool for exploratory analysis. Overall, this comparative evaluation provides practical guidance for selecting high-dimensional mediation methods in large-scale epigenetic studies.

## Supplementary Material

vbag331_Supplementary_Data

## Data Availability

The participant-level data supporting the findings of this study are available upon application to the Taiwan Biobank. To access the data, applicants should submit a request via the official website at https://www.twbiobank.org.tw/. The HDMTS code is available at https://github.com/WanYuLin/HDMTS, and the simulation code is available at https://github.com/WanYuLin/HDMTS/tree/main/Simulations.
