# Supplementary material for: Benchmarking high-dimensional mediation methods in epigenome-wide studies
Source: Bioinform Adv. 2026 Oct 30;6(1):vbag331. doi: 10.1093/bioadv/vbag331 (PMC13770479; doi:10.1093/bioadv/vbag331)
Supplement: Supplementary file 1 [file vbag331_supplementary_data.pdf]

# Supplementary Materials of

# Benchmarking High-Dimensional Mediation Methods in Epigenome-Wide Studies

**Wan-Yu Lin [1,2*]**

[1] Institute of Health Data Analytics and Statistics, College of Public Health, National Taiwan University, Taipei, Taiwan
[2] Master of Public Health Program, College of Public Health, National Taiwan University, Taipei, Taiwan

*** Correspondence:**
Corresponding Author: Wan-Yu Lin, Ph.D. (https://orcid.org/0000-0002-3385-4702)
E-mail: linwy@ntu.edu.tw
Room 501, No. 17, Xu-Zhou Road, Taipei 100, Taiwan
Phone/Fax: +886-2-33668106

## Contents

Table S1. Percentage of simulation replications with no mediators identified by each method under each simulation setting. ........ 4
Table S2. Block-structured correlation scenario: Percentage of simulation replications with no mediators identified by each method under each simulation setting. ........ 7
Figure S1. Simulation results under the simulation setting using one-third of the $\boldsymbol{\alpha_j}$ values ($j$ = 1, …, $p$) ........ 8
Figure S2. Bias of estimated mediation effects under the simulation setting using one-third of the $\boldsymbol{\alpha_j}$ values ($j$ = 1, …, $p$) ........ 10
Figure S3. Simulation results under the simulation setting using one-third of the $\boldsymbol{\beta_j}$ values ($j$ = 1, …, $p$) ........ 11
Figure S4. Bias of estimated mediation effects under the simulation setting using one-third of the $\boldsymbol{\beta_j}$ values ($j$ = 1, …, $p$) ........ 12
Figure S5. Block-structured correlation scenario: Simulation results under the simulation setting based on the $\boldsymbol{\alpha_j}$ and $\boldsymbol{\beta_j}$ values reported by Perera *et al.* ($j$ = 1, …, $p$) ........ 14
Figure S6. Block-structured correlation scenario: Bias of estimated mediation effects under the simulation setting based on the $\boldsymbol{\alpha_j}$ and $\boldsymbol{\beta_j}$ values from Perera *et al.* ($j$ = 1, …, $p$) ........ 15
Figure S7. Block-structured correlation scenario: Simulation results under the simulation setting using half of the $\boldsymbol{\alpha_j}$ values ($j$ = 1, …, $p$) ........ 17
Figure S8. Block-structured correlation scenario: Bias of estimated mediation effects under the simulation setting using half of the $\boldsymbol{\alpha_j}$ values ($j$ = 1, …, $p$) ........ 18
Figure S9. Block-structured correlation scenario: Simulation results under the simulation setting using half of the $\boldsymbol{\beta_j}$ values ($j$ = 1, …, $p$) ........ 20
Figure S10. Block-structured correlation scenario: Bias of estimated mediation effects under the simulation setting using half of the $\boldsymbol{\beta_j}$ values ($j$ = 1, …, $p$) ........ 21
Figure S11. Block-structured correlation scenario: Simulation results under the simulation setting using one-third of the $\boldsymbol{\alpha_j}$ values ($j$ = 1, …, $p$) ........ 23
Figure S12. Block-structured correlation scenario: Bias of estimated mediation effects under the simulation setting using one-third of the $\boldsymbol{\alpha_j}$ values ($j$ = 1, …, $p$) ........ 24

Figure S13. Simulation results under the simulation setting using one-third of the $\boldsymbol{\beta j}$ values ($j$ = 1, …, $p$) ...... 26
Figure S14. Block-structured correlation scenario: Bias of estimated mediation effects under the simulation setting using one-third of the $\boldsymbol{\beta j}$ values ($j$ = 1, …, $p$) ........................................................................ 27

| Condition | *N* | *p* | *ρ* | HIMA | HIMA2 | HDMA | HDMTS |
|---|---|---|---|---|---|---|---|
| **Perera *et al.*'s setting** | 300 | 1000 | 0 | 0.10% | 0.00% | 0.00% | 0.00% |
| | 300 | 1000 | 0.25 | 0.00% | 0.00% | 0.00% | 0.00% |
| | 300 | 1000 | 0.5 | 0.00% | 0.00% | 0.00% | 0.00% |
| | 300 | 1000 | 0.75 | 0.20% | 0.00% | 0.00% | 0.00% |
| | 300 | 5000 | 0 | 3.20% | 0.00% | 0.30% | 0.00% |
| | 300 | 5000 | 0.25 | 0.80% | 0.00% | 0.20% | 0.00% |
| | 300 | 5000 | 0.5 | 0.70% | 0.10% | 0.00% | 0.00% |
| | 300 | 5000 | 0.75 | 0.60% | 0.50% | 0.20% | 0.00% |
| | 600 | 1000 | 0 | 0.00% | 0.00% | 0.00% | 0.00% |
| | 600 | 1000 | 0.25 | 0.00% | 0.00% | 0.00% | 0.00% |
| | 600 | 1000 | 0.5 | 0.00% | 0.00% | 0.00% | 0.00% |
| | 600 | 1000 | 0.75 | 0.00% | 0.00% | 0.00% | 0.00% |
| | 600 | 5000 | 0 | 0.00% | 0.00% | 0.00% | 0.00% |
| | 600 | 5000 | 0.25 | 0.00% | 0.00% | 0.00% | 0.00% |
| | 600 | 5000 | 0.5 | 0.00% | 0.00% | 0.00% | 0.00% |
| | 600 | 5000 | 0.75 | 0.00% | 0.00% | 0.00% | 0.00% |
| **Alpha/2** | 300 | 1000 | 0 | 5.50% | 3.80% | 2.80% | 0.50% |
| | 300 | 1000 | 0.25 | 4.90% | 7.20% | 3.50% | 0.80% |
| | 300 | 1000 | 0.5 | 5.10% | 11.20% | 4.60% | 2.00% |
| | 300 | 1000 | 0.75 | 9.20% | 20.10% | 6.20% | 4.20% |
| | 300 | 5000 | 0 | 14.90% | 4.80% | 7.80% | 1.60% |
| | 300 | 5000 | 0.25 | 12.20% | 5.50% | 6.40% | 1.20% |
| | 300 | 5000 | 0.5 | 13.50% | 10.90% | 8.80% | 3.30% |
| | 300 | 5000 | 0.75 | 19.30% | 22.30% | 11.70% | 7.80% |
| | 600 | 1000 | 0 | 0.00% | 0.00% | 0.00% | 0.00% |
| | 600 | 1000 | 0.25 | 0.00% | 0.00% | 0.00% | 0.00% |
| | 600 | 1000 | 0.5 | 0.00% | 0.10% | 0.00% | 0.00% |
| | 600 | 1000 | 0.75 | 0.30% | 0.60% | 0.00% | 0.00% |
| | 600 | 5000 | 0 | 0.00% | 0.00% | 0.00% | 0.00% |
| | 600 | 5000 | 0.25 | 0.00% | 0.00% | 0.00% | 0.00% |
| | 600 | 5000 | 0.5 | 0.00% | 0.20% | 0.00% | 0.00% |
| | 600 | 5000 | 0.75 | 0.10% | 0.60% | 0.10% | 0.20% |
| **Alpha/3** | 300 | 1000 | 0 | 42.20% | 48.00% | 40.90% | 17.50% |
| | 300 | 1000 | 0.25 | 40.80% | 56.00% | 43.60% | 21.20% |
| | 300 | 1000 | 0.5 | 37.90% | 63.40% | 42.90% | 25.40% |
| | 300 | 1000 | 0.75 | 45.10% | 70.60% | 43.80% | 35.20% |

| Condition | *N* | *p* | *ρ* | HIMA | HIMA2 | HDMA | HDMTS |
|---|---|---|---|---|---|---|---|
| | 300 | 5000 | 0 | 58.50% | 47.50% | 52.10% | 30.80% |
| | 300 | 5000 | 0.25 | 56.90% | 48.50% | 48.40% | 30.00% |
| | 300 | 5000 | 0.5 | 60.10% | 56.60% | 51.60% | 34.40% |
| | 300 | 5000 | 0.75 | 59.00% | 67.00% | 50.40% | 41.80% |
| | 600 | 1000 | 0 | 0.60% | 10.10% | 4.60% | 0.60% |
| | 600 | 1000 | 0.25 | 0.90% | 14.00% | 5.60% | 1.00% |
| | 600 | 1000 | 0.5 | 1.60% | 21.50% | 8.60% | 2.50% |
| | 600 | 1000 | 0.75 | 7.50% | 30.40% | 10.90% | 5.40% |
| | 600 | 5000 | 0 | 7.40% | 6.60% | 6.00% | 1.40% |
| | 600 | 5000 | 0.25 | 7.60% | 11.50% | 8.80% | 1.50% |
| | 600 | 5000 | 0.5 | 6.10% | 17.00% | 9.80% | 5.20% |
| | 600 | 5000 | 0.75 | 10.50% | 28.60% | 16.00% | 11.60% |
| **Beta/2** | 300 | 1000 | 0 | 36.50% | 15.90% | 22.20% | 12.70% |
| | 300 | 1000 | 0.25 | 15.90% | 14.40% | 16.20% | 0.60% |
| | 300 | 1000 | 0.5 | 7.30% | 20.30% | 17.80% | 0.00% |
| | 300 | 1000 | 0.75 | 3.80% | 39.90% | 16.90% | 0.00% |
| | 300 | 5000 | 0 | 59.40% | 25.50% | 46.10% | 22.40% |
| | 300 | 5000 | 0.25 | 34.20% | 21.80% | 31.70% | 2.20% |
| | 300 | 5000 | 0.5 | 18.70% | 28.30% | 37.00% | 0.00% |
| | 300 | 5000 | 0.75 | 14.80% | 44.80% | 39.80% | 0.00% |
| | 600 | 1000 | 0 | 11.90% | 1.10% | 2.20% | 0.20% |
| | 600 | 1000 | 0.25 | 0.40% | 1.20% | 1.40% | 0.00% |
| | 600 | 1000 | 0.5 | 0.00% | 2.30% | 1.10% | 0.00% |
| | 600 | 1000 | 0.75 | 0.10% | 15.50% | 2.20% | 0.00% |
| | 600 | 5000 | 0 | 18.90% | 3.70% | 11.80% | 0.80% |
| | 600 | 5000 | 0.25 | 6.80% | 2.80% | 9.50% | 0.00% |
| | 600 | 5000 | 0.5 | 3.00% | 5.70% | 12.70% | 0.00% |
| | 600 | 5000 | 0.75 | 0.80% | 20.50% | 15.90% | 0.00% |
| **Beta/3** | 300 | 1000 | 0 | 69.10% | 55.70% | 57.60% | 50.80% |
| | 300 | 1000 | 0.25 | 52.30% | 52.10% | 52.60% | 20.20% |
| | 300 | 1000 | 0.5 | 26.90% | 61.00% | 54.60% | 2.70% |
| | 300 | 1000 | 0.75 | 11.60% | 73.40% | 49.60% | 0.20% |
| | 300 | 5000 | 0 | 82.50% | 58.20% | 75.50% | 63.60% |
| | 300 | 5000 | 0.25 | 63.70% | 54.80% | 62.70% | 28.20% |
| | 300 | 5000 | 0.5 | 44.30% | 62.70% | 65.20% | 4.20% |
| | 300 | 5000 | 0.75 | 30.40% | 74.80% | 69.00% | 0.00% |
| | 600 | 1000 | 0 | 59.40% | 27.10% | 30.40% | 16.20% |
| | 600 | 1000 | 0.25 | 19.80% | 24.30% | 24.20% | 1.20% |
| | 600 | 1000 | 0.5 | 0.80% | 38.40% | 28.90% | 0.00% |

| Condition | *N* | *p* | *ρ* | HIMA | HIMA2 | HDMA | HDMTS |
|---|---|---|---|---|---|---|---|
| | 600 | 1000 | 0.75 | 0.20% | 56.30% | 28.00% | 0.00% |
| | 600 | 5000 | 0 | 57.10% | 37.30% | 54.00% | 27.60% |
| | 600 | 5000 | 0.25 | 35.00% | 37.00% | 43.60% | 1.70% |
| | 600 | 5000 | 0.5 | 15.50% | 46.30% | 50.20% | 0.00% |
| | 600 | 5000 | 0.75 | 7.30% | 63.80% | 55.70% | 0.00% |

**Table S1. Percentage of simulation replications with no mediators identified by each method under each simulation setting.**

Values represent the percentage of simulation replications in which a method identified no mediators. In these replications, the false discovery proportion (FDP) was set to zero. The mean FDP was calculated across all simulation replications to estimate the false discovery rate (FDR). *N* denotes the sample size, *p* the number of candidate mediators, and ρ the AR(1) correlation parameter for the mediator error terms.

| Condition | *N* | *p* | *ρ* | HIMA | HIMA2 | HDMA | HDMTS |
|---|---|---|---|---|---|---|---|
| **Perera *et al.*'s setting** | 300 | 1000 | 0 | 0.20% | 0.00% | 0.00% | 0.00% |
| | 300 | 1000 | 0.25 | 0.00% | 0.00% | 0.00% | 0.00% |
| | 300 | 1000 | 0.5 | 0.00% | 0.00% | 0.00% | 0.00% |
| | 300 | 1000 | 0.75 | 0.10% | 0.10% | 0.00% | 0.00% |
| | 300 | 5000 | 0 | 2.70% | 0.00% | 0.60% | 0.00% |
| | 300 | 5000 | 0.25 | 0.80% | 0.00% | 0.10% | 0.00% |
| | 300 | 5000 | 0.5 | 0.80% | 0.00% | 0.00% | 0.00% |
| | 300 | 5000 | 0.75 | 0.80% | 0.10% | 0.10% | 0.00% |
| | 600 | 1000 | 0 | 0.00% | 0.00% | 0.00% | 0.00% |
| | 600 | 1000 | 0.25 | 0.00% | 0.00% | 0.00% | 0.00% |
| | 600 | 1000 | 0.5 | 0.00% | 0.00% | 0.00% | 0.00% |
| | 600 | 1000 | 0.75 | 0.00% | 0.00% | 0.00% | 0.00% |
| | 600 | 5000 | 0 | 0.00% | 0.00% | 0.00% | 0.00% |
| | 600 | 5000 | 0.25 | 0.00% | 0.00% | 0.00% | 0.00% |
| | 600 | 5000 | 0.5 | 0.00% | 0.00% | 0.00% | 0.00% |
| | 600 | 5000 | 0.75 | 0.00% | 0.00% | 0.00% | 0.00% |
| **Alpha/2** | 300 | 1000 | 0 | 5.50% | 4.70% | 2.50% | 0.70% |
| | 300 | 1000 | 0.25 | 4.60% | 6.90% | 3.70% | 1.10% |
| | 300 | 1000 | 0.5 | 5.60% | 12.20% | 4.90% | 1.30% |
| | 300 | 1000 | 0.75 | 8.60% | 20.80% | 6.60% | 3.20% |
| | 300 | 5000 | 0 | 15.50% | 4.20% | 6.70% | 1.80% |
| | 300 | 5000 | 0.25 | 13.50% | 6.10% | 7.40% | 1.80% |
| | 300 | 5000 | 0.5 | 13.10% | 10.10% | 8.10% | 2.80% |
| | 300 | 5000 | 0.75 | 20.20% | 21.30% | 12.20% | 7.80% |
| | 600 | 1000 | 0 | 0.00% | 0.00% | 0.00% | 0.00% |
| | 600 | 1000 | 0.25 | 0.00% | 0.00% | 0.00% | 0.00% |
| | 600 | 1000 | 0.5 | 0.00% | 0.10% | 0.00% | 0.00% |
| | 600 | 1000 | 0.75 | 0.10% | 0.40% | 0.00% | 0.00% |
| | 600 | 5000 | 0 | 0.00% | 0.00% | 0.00% | 0.00% |
| | 600 | 5000 | 0.25 | 0.00% | 0.00% | 0.00% | 0.00% |
| | 600 | 5000 | 0.5 | 0.10% | 0.10% | 0.10% | 0.10% |
| | 600 | 5000 | 0.75 | 0.20% | 0.20% | 0.00% | 0.00% |
| **Alpha/3** | 300 | 1000 | 0 | 43.60% | 48.70% | 40.20% | 18.80% |
| | 300 | 1000 | 0.25 | 39.50% | 53.70% | 41.00% | 20.40% |
| | 300 | 1000 | 0.5 | 37.80% | 60.30% | 42.50% | 25.60% |
| | 300 | 1000 | 0.75 | 43.80% | 69.10% | 43.90% | 35.90% |
| | 300 | 5000 | 0 | 55.90% | 43.60% | 47.10% | 26.70% |
| | 300 | 5000 | 0.25 | 58.30% | 52.40% | 51.00% | 32.50% |

| | | | | | | | |
|---|---|---|---|---|---|---|---|
| | 300 | 5000 | 0.5 | 57.40% | 56.90% | 50.40% | 35.00% |
| | 300 | 5000 | 0.75 | 60.10% | 69.00% | 52.20% | 44.20% |
| | 600 | 1000 | 0 | 0.30% | 10.40% | 4.60% | 0.40% |
| | 600 | 1000 | 0.25 | 0.80% | 12.70% | 4.80% | 1.40% |
| | 600 | 1000 | 0.5 | 2.00% | 21.20% | 8.20% | 2.80% |
| | 600 | 1000 | 0.75 | 7.20% | 28.00% | 9.80% | 6.80% |
| | 600 | 5000 | 0 | 9.00% | 7.30% | 6.80% | 1.30% |
| | 600 | 5000 | 0.25 | 8.00% | 11.90% | 9.80% | 4.20% |
| | 600 | 5000 | 0.5 | 6.00% | 18.50% | 11.00% | 4.90% |
| | 600 | 5000 | 0.75 | 9.50% | 26.00% | 11.60% | 8.60% |
| **Beta/2** | 300 | 1000 | 0 | 34.50% | 16.50% | 21.60% | 12.40% |
| | 300 | 1000 | 0.25 | 18.70% | 15.70% | 19.10% | 0.60% |
| | 300 | 1000 | 0.5 | 7.10% | 21.20% | 17.80% | 0.00% |
| | 300 | 1000 | 0.75 | 5.10% | 38.90% | 17.50% | 0.00% |
| | 300 | 5000 | 0 | 56.80% | 25.70% | 41.30% | 21.10% |
| | 300 | 5000 | 0.25 | 35.50% | 20.30% | 30.10% | 2.40% |
| | 300 | 5000 | 0.5 | 19.80% | 27.00% | 37.20% | 0.00% |
| | 300 | 5000 | 0.75 | 14.20% | 44.40% | 37.90% | 0.00% |
| | 600 | 1000 | 0 | 11.50% | 1.10% | 2.40% | 0.20% |
| | 600 | 1000 | 0.25 | 0.70% | 0.70% | 1.80% | 0.00% |
| | 600 | 1000 | 0.5 | 0.00% | 2.60% | 0.40% | 0.00% |
| | 600 | 1000 | 0.75 | 0.00% | 15.10% | 2.10% | 0.00% |
| | 600 | 5000 | 0 | 18.40% | 3.50% | 11.20% | 0.90% |
| | 600 | 5000 | 0.25 | 8.60% | 2.30% | 8.50% | 0.00% |
| | 600 | 5000 | 0.5 | 2.30% | 5.40% | 10.60% | 0.00% |
| | 600 | 5000 | 0.75 | 0.90% | 20.70% | 16.70% | 0.00% |
| **Beta/3** | 300 | 1000 | 0 | 63.90% | 54.40% | 58.60% | 48.30% |
| | 300 | 1000 | 0.25 | 50.70% | 53.70% | 52.90% | 19.20% |
| | 300 | 1000 | 0.5 | 24.30% | 61.40% | 57.20% | 2.20% |
| | 300 | 1000 | 0.75 | 10.80% | 72.80% | 51.10% | 0.10% |
| | 300 | 5000 | 0 | 80.40% | 60.20% | 73.90% | 63.70% |
| | 300 | 5000 | 0.25 | 66.90% | 57.90% | 62.30% | 28.80% |
| | 300 | 5000 | 0.5 | 46.40% | 67.80% | 67.80% | 4.80% |
| | 300 | 5000 | 0.75 | 31.00% | 74.20% | 67.10% | 0.10% |
| | 600 | 1000 | 0 | 59.50% | 28.60% | 33.20% | 15.90% |
| | 600 | 1000 | 0.25 | 20.20% | 26.70% | 28.70% | 1.20% |
| | 600 | 1000 | 0.5 | 1.10% | 35.00% | 26.60% | 0.00% |
| | 600 | 1000 | 0.75 | 0.10% | 58.90% | 28.30% | 0.00% |
| | 600 | 5000 | 0 | 56.20% | 37.60% | 49.10% | 24.90% |
| | 600 | 5000 | 0.25 | 31.60% | 33.80% | 42.30% | 2.60% |

| | | | | | | | |
|---|---|---|---|---|---|---|---|
| | 600 | 5000 | 0.5 | 15.90% | 45.20% | 51.60% | 0.00% |
| | 600 | 5000 | 0.75 | 7.40% | 66.40% | 57.60% | 0.00% |

**Table S2. Block-structured correlation scenario: Percentage of simulation replications with no mediators identified by each method under each simulation setting.**

Values represent the percentage of simulation replications in which a method identified no mediators. In these replications, the false discovery proportion (FDP) was set to zero. The mean FDP was calculated across all simulation replications to estimate the false discovery rate (FDR). *N* denotes the sample size, *p* the number of candidate mediators, and ρ the AR(1) correlation parameter for the mediator error terms.

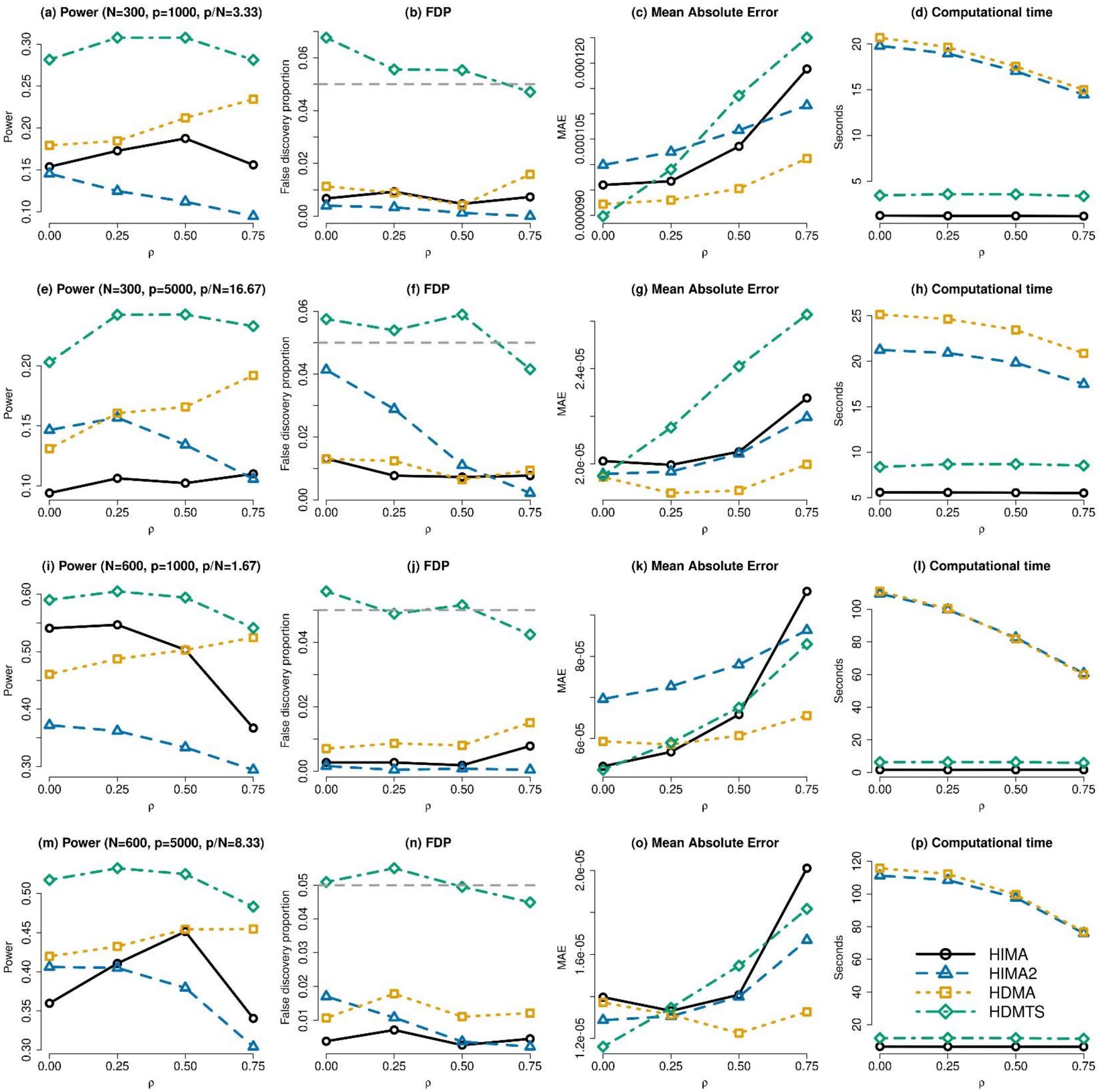


Figure S1. **Simulation results under the simulation setting using one-third of the $\alpha_j$ values (*j* = 1, …, *p*)**

Results are averaged over 1,000 simulation replications. The columns (from left to right) show statistical power, false discovery proportion (FDP), mean absolute error (MAE) of the estimated mediation effect, and computational time (seconds). The rows (from top to bottom) correspond to the settings: *N*=300, *p*=1000; *N*=300, *p*=5000; *N*=600, *p*=1000; and *N*=600, *p*=5000. The *x*-axis denotes correlation levels among CpG sites

($\rho = 0, 0.25, 0.50, or\ 0.75$). The gray horizontal line in the FDP panels indicates the nominal FDR level of 0.05.

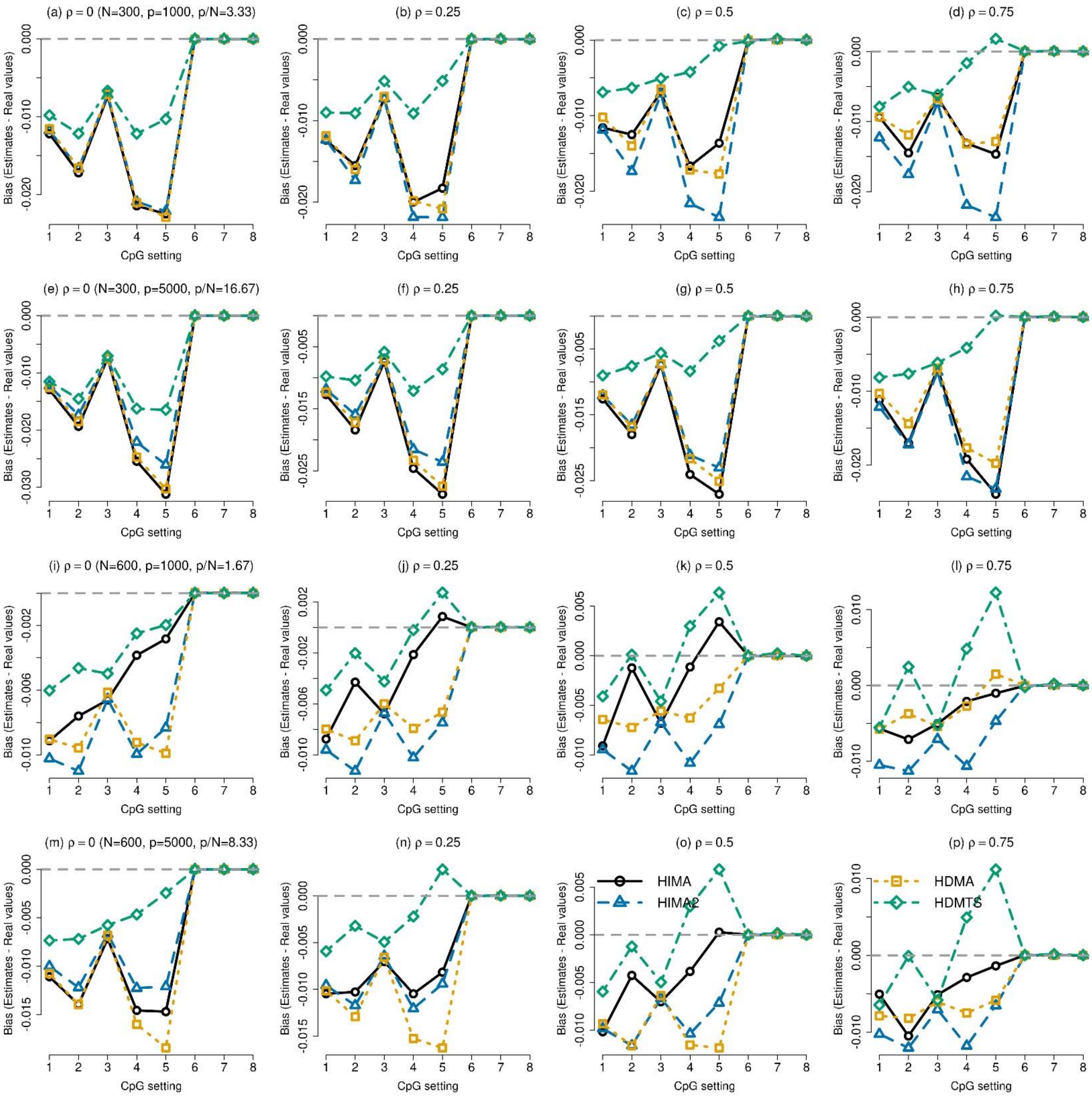

Figure S2. **Bias of estimated mediation effects under the simulation setting using one-third of the $\alpha_j$ values (*j* = 1, …, *p*)**

The rows (from top to bottom) correspond to the settings: *N*=300, *p*=1000; *N*=300, *p*=5000; *N*=600, *p*=1000; and *N*=600, *p*=5000. The columns (from left to right) represent correlation levels among CpG sites ($\rho = 0, 0.25, 0.50, or\ 0.75$). Bias was calculated as the average difference between estimated and true mediation effects over 1,000 simulation replications. The *x*-axis denotes CpG settings corresponding to true nonzero effects: $\alpha_1 = 0.067$, $\alpha_2 = 0.083$, $\alpha_3 = 0.05$, $\alpha_4 = 0.10$, $\alpha_5 = 0.117$, $\alpha_7 = 0.033$, and $\alpha_j = 0$ otherwise; $\beta_1 = 0.20$, $\beta_2 = 0.25$, $\beta_3 = 0.15$, $\beta_4 = 0.30$, $\beta_5 = 0.35$, $\beta_6 = 0.10$, and $\beta_j = 0$ otherwise. CpG setting 8 represents the average bias across all CpG sites with $j \geq 8$. The gray horizontal lines indicate zero bias.

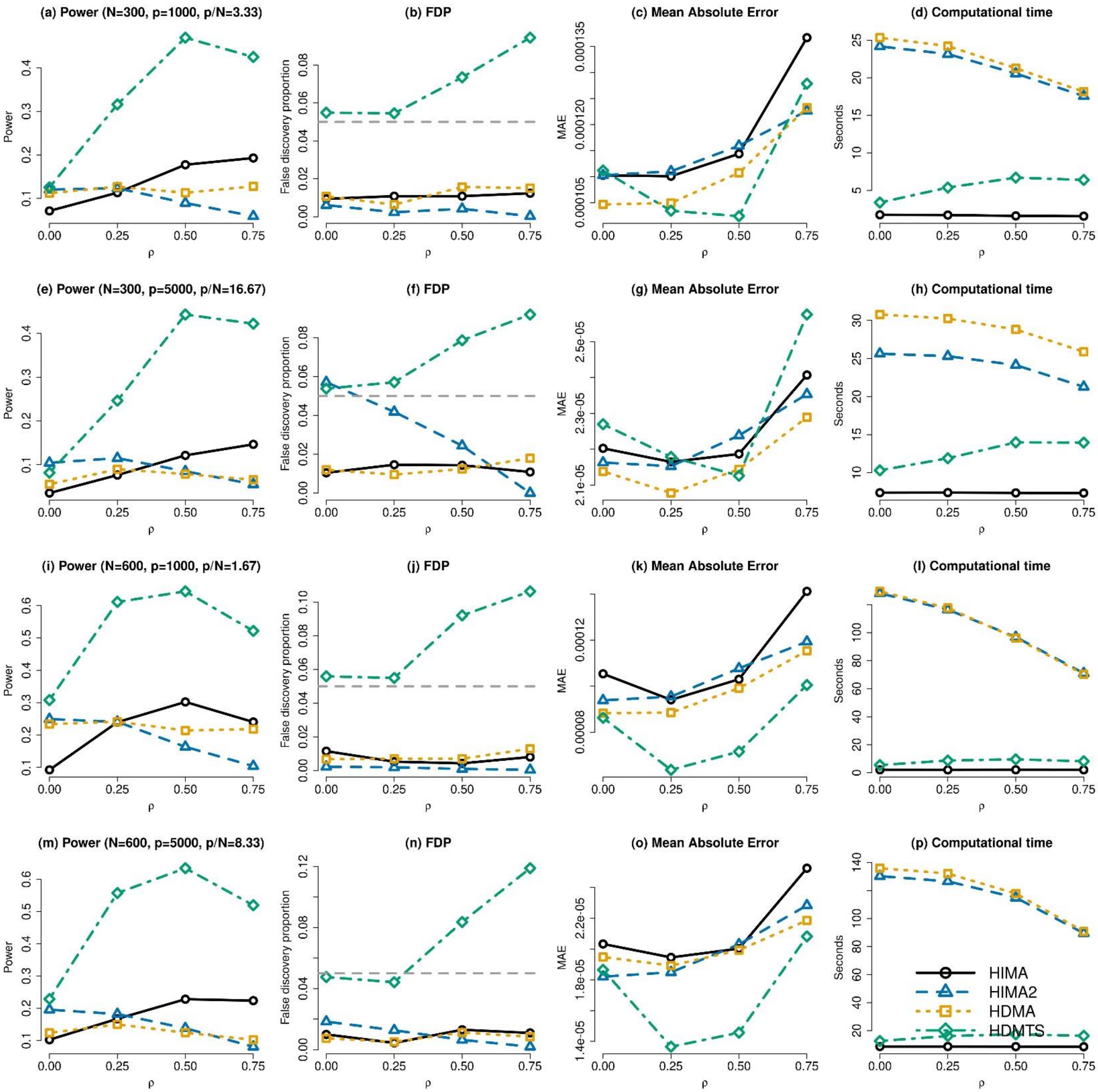


Figure S3. **Simulation results under the simulation setting using one-third of the $\boldsymbol{\beta_j}$ values (*j* = 1, …, *p*)**

Results are averaged over 1,000 simulation replications. The columns (from left to right) show statistical power, false discovery proportion (FDP), mean absolute error (MAE) of the estimated mediation effect, and computational time (seconds). The rows (from top to bottom) correspond to the settings: *N*=300, *p*=1000; *N*=300, *p*=5000; *N*=600, *p*=1000; and *N*=600, *p*=5000. The *x*-axis denotes correlation levels among CpG sites

($\rho = 0, 0.25, 0.50, or\ 0.75$). The gray horizontal line in the FDP panels indicates the nominal FDR level of 0.05.

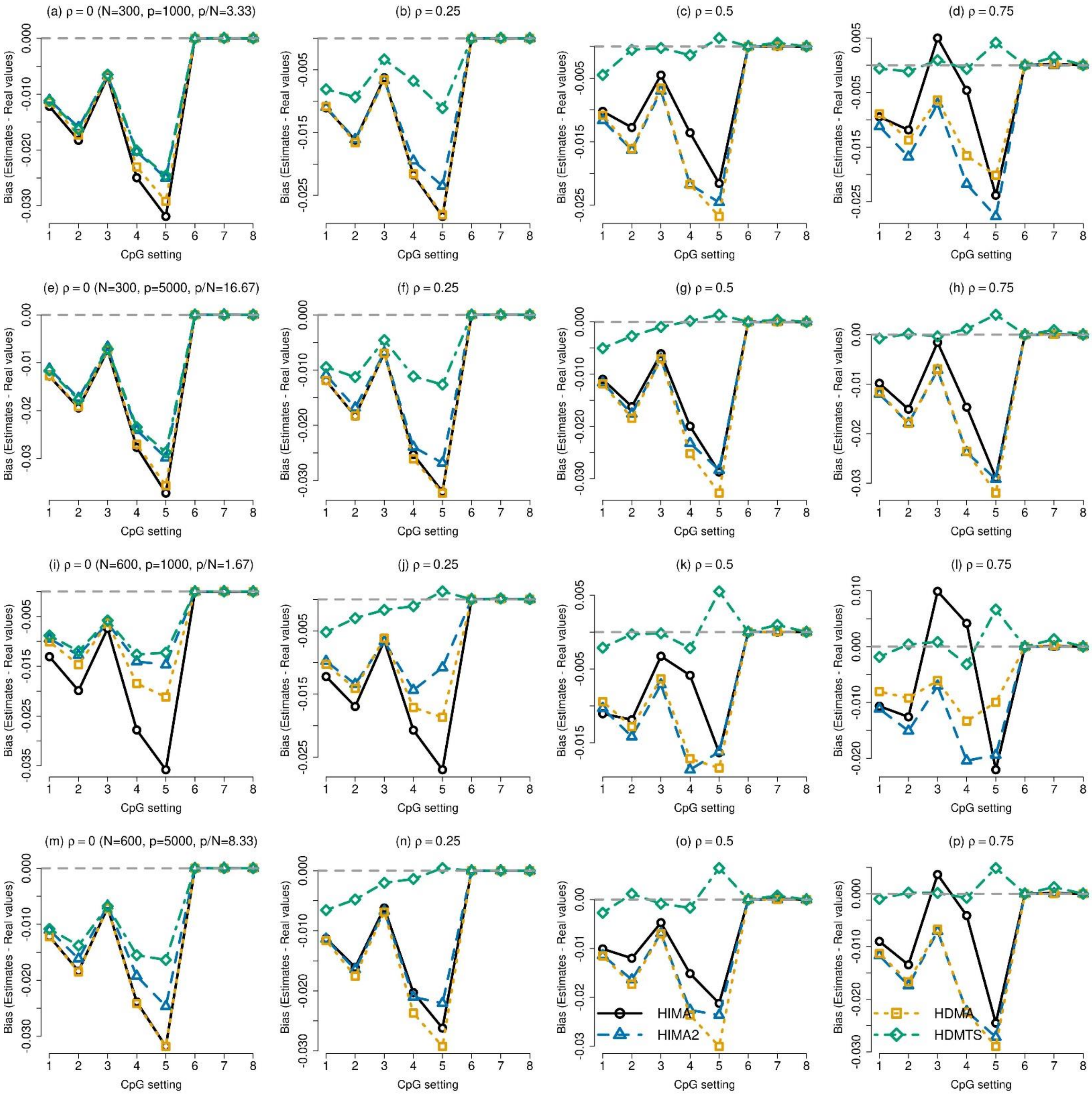


Figure S4. **Bias of estimated mediation effects under the simulation setting using one-third of the $\boldsymbol{\beta_j}$ values ($\boldsymbol{j}$ = 1, …, $\boldsymbol{p}$)**

The rows (from top to bottom) correspond to the settings: *N*=300, *p*=1000; *N*=300, *p*=5000; *N*=600, *p*=1000;

and $N$=600, $p$=5000. The columns (from left to right) represent correlation levels among CpG sites ($\rho = 0, 0.25, 0.50, or\ 0.75$). Bias was calculated as the average difference between estimated and true mediation effects over 1,000 simulation replications. The $x$-axis denotes CpG settings corresponding to true nonzero effects: $\alpha_1 = 0.20$, $\alpha_2 = 0.25$, $\alpha_3 = 0.15$, $\alpha_4 = 0.30$, $\alpha_5 = 0.35$, $\alpha_7 = 0.10$, and $\alpha_j = 0$ otherwise; $\beta_1 = 0.067$, $\beta_2 = 0.083$, $\beta_3 = 0.05$, $\beta_4 = 0.10$, $\beta_5 = 0.117$, $\beta_6 = 0.033$, and $\beta_j = 0$ otherwise. CpG setting 8 represents the average bias across all CpG sites with $j \geq 8$. The gray horizontal lines indicate zero bias.

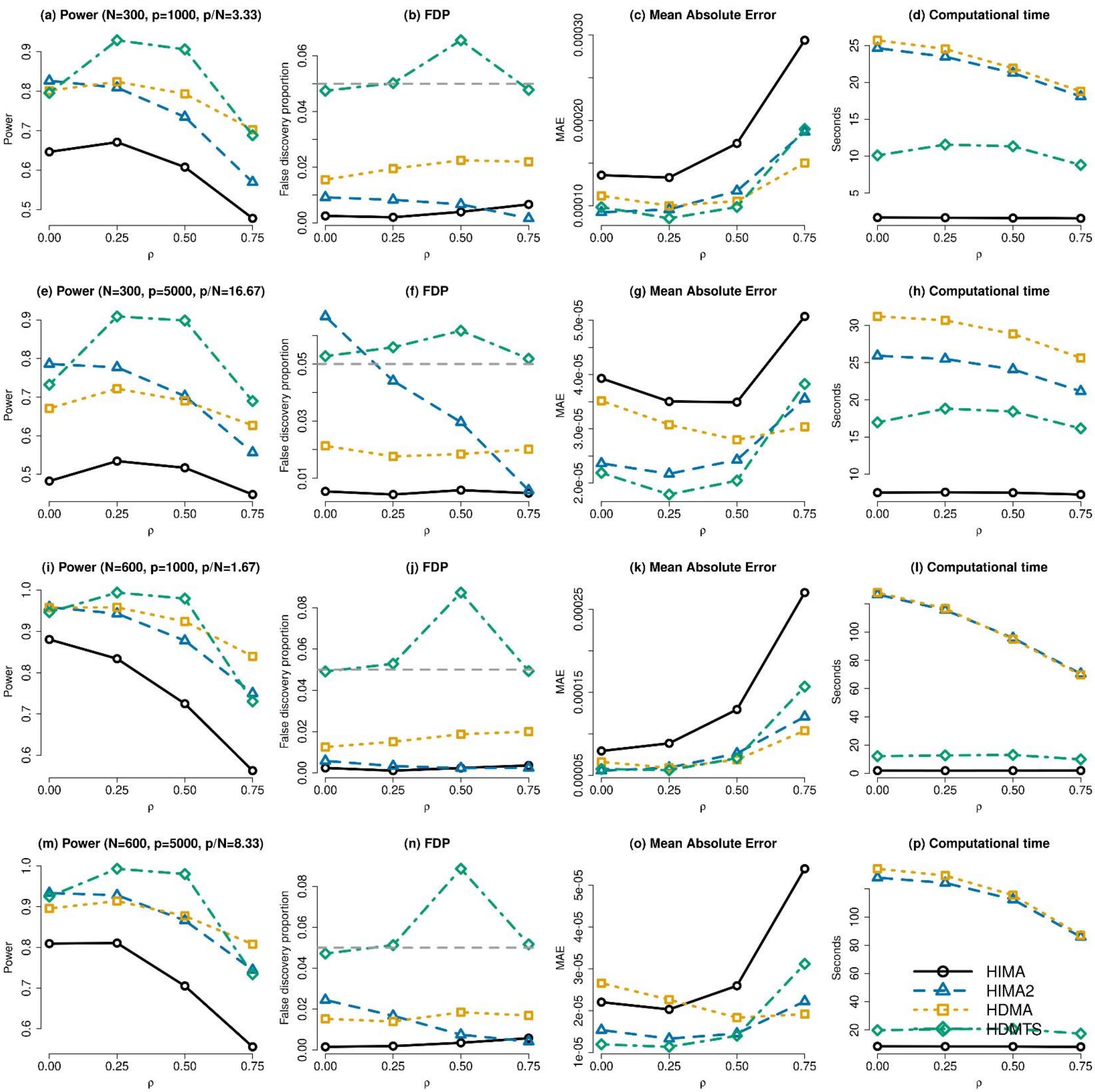

Figure S5. **Block-structured correlation scenario: Simulation results under the simulation setting based on the $\alpha_j$ and $\beta_j$ values reported by Perera *et al*. (*j* = 1, …, *p*)**

Results are averaged over 1,000 simulation replications. The columns (from left to right) show statistical power, false discovery proportion (FDP), mean absolute error (MAE) of the estimated mediation effect, and computational time (seconds). The rows (from top to bottom) correspond to the settings: *N*=300, *p*=1000; *N*=300, *p*=5000; *N*=600, *p*=1000; and *N*=600, *p*=5000. The *x*-axis denotes correlation levels among CpG sites ($\rho = 0, 0.25, 0.50, or\ 0.75$). The gray horizontal line in the FDP panels indicates the nominal FDR level of 0.05.

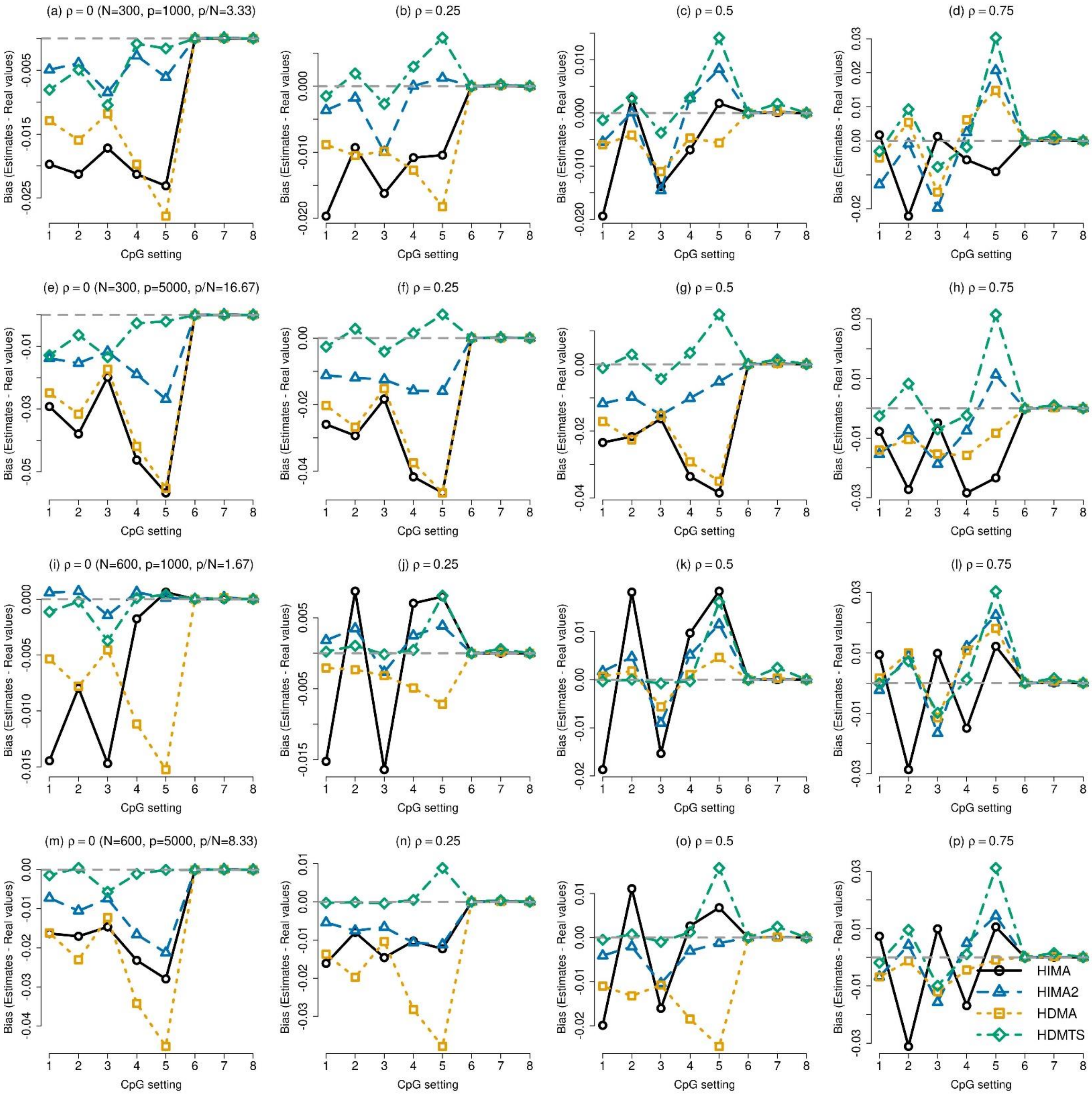


Figure S6. **Block-structured correlation scenario: Bias of estimated mediation effects under the simulation setting based on the $\alpha_j$ and $\beta_j$ values from Perera *et al.* (*j* = 1, …, *p*)**

The rows (from top to bottom) correspond to the settings: *N*=300, *p*=1000; *N*=300, *p*=5000; *N*=600, *p*=1000; and *N*=600, *p*=5000. The columns (from left to right) represent correlation levels among CpG sites ($\rho = 0, 0.25, 0.50, or\ 0.75$). Bias was calculated as the average difference between estimated and true mediation

effects over 1,000 simulation replications. The $x$-axis denotes CpG settings corresponding to true nonzero effects: $\alpha_1 = 0.20$, $\alpha_2 = 0.25$, $\alpha_3 = 0.15$, $\alpha_4 = 0.30$, $\alpha_5 = 0.35$, $\alpha_7 = 0.10$, and $\alpha_j = 0$ otherwise; $\beta_1 = 0.20$, $\beta_2 = 0.25$, $\beta_3 = 0.15$, $\beta_4 = 0.30$, $\beta_5 = 0.35$, $\beta_6 = 0.10$, and $\beta_j = 0$ otherwise. CpG setting 8 represents the average bias across all CpG sites with $j \geq 8$. The gray horizontal lines indicate zero bias.

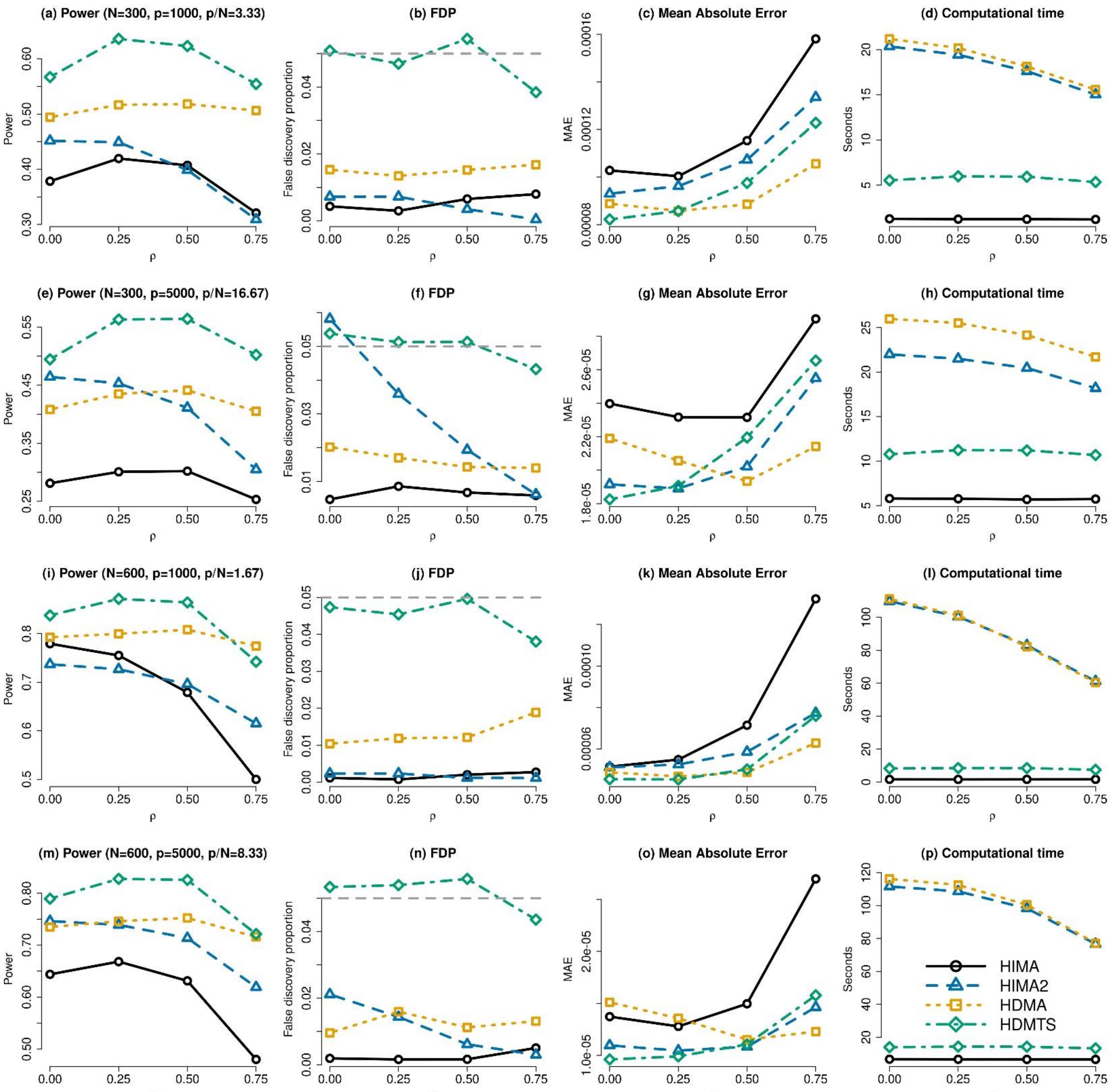

Figure S7. **Block-structured correlation scenario: Simulation results under the simulation setting using half of the $\boldsymbol{\alpha_j}$ values (*j* = 1, …, *p*)**

Results are averaged over 1,000 simulation replications. The columns (from left to right) show statistical power, false discovery proportion (FDP), mean absolute error (MAE) of the estimated mediation effect, and computational time (seconds). The rows (from top to bottom) correspond to the settings: $N$=300, $p$=1000; $N$=300, $p$=5000; $N$=600, $p$=1000; and $N$=600, $p$=5000. The $x$-axis denotes correlation levels among CpG sites ($\rho = 0, 0.25, 0.50, or\ 0.75$). The gray horizontal line in the FDP panels indicates the nominal FDR level of 0.05.

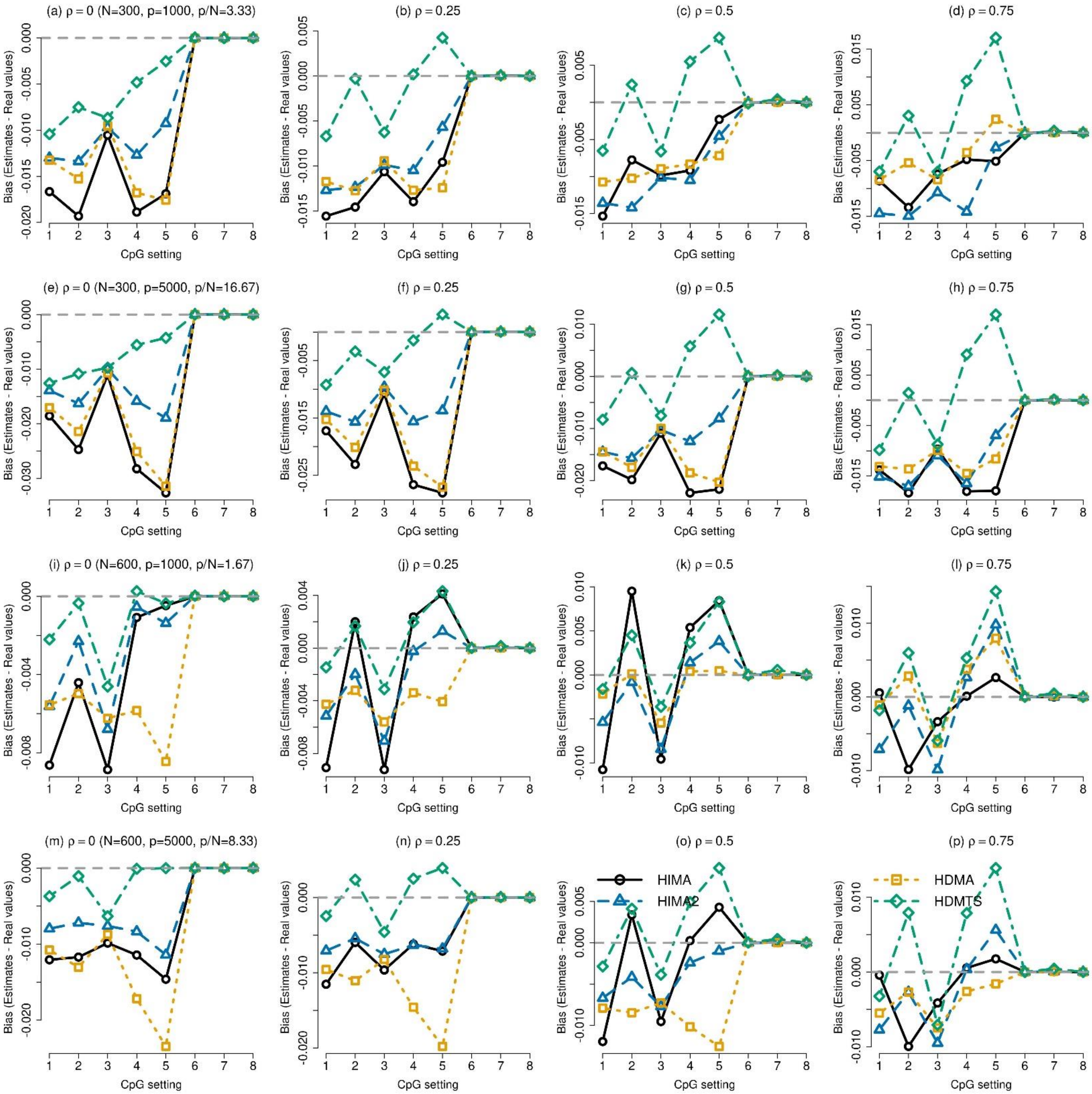


Figure S8. **Block-structured correlation scenario: Bias of estimated mediation effects under the simulation setting using half of the $\alpha_j$ values ($j$ = 1, …, $p$)**

The rows (from top to bottom) correspond to the settings: *N*=300, *p*=1000; *N*=300, *p*=5000; *N*=600, *p*=1000; and *N*=600, *p*=5000. The columns (from left to right) represent correlation levels among CpG sites ($\rho = 0, 0.25, 0.50, or\ 0.75$). Bias was calculated as the average difference between estimated and true mediation

effects over 1,000 simulation replications. The $x$-axis denotes CpG settings corresponding to true nonzero effects: $\alpha_1 = 0.10$, $\alpha_2 = 0.125$, $\alpha_3 = 0.075$, $\alpha_4 = 0.15$, $\alpha_5 = 0.175$, $\alpha_7 = 0.05$, and $\alpha_j = 0$ otherwise; $\beta_1 = 0.20$, $\beta_2 = 0.25$, $\beta_3 = 0.15$, $\beta_4 = 0.30$, $\beta_5 = 0.35$, $\beta_6 = 0.10$, and $\beta_j = 0$ otherwise. CpG setting 8 represents the average bias across all CpG sites with $j \geq 8$. The gray horizontal lines indicate zero bias.

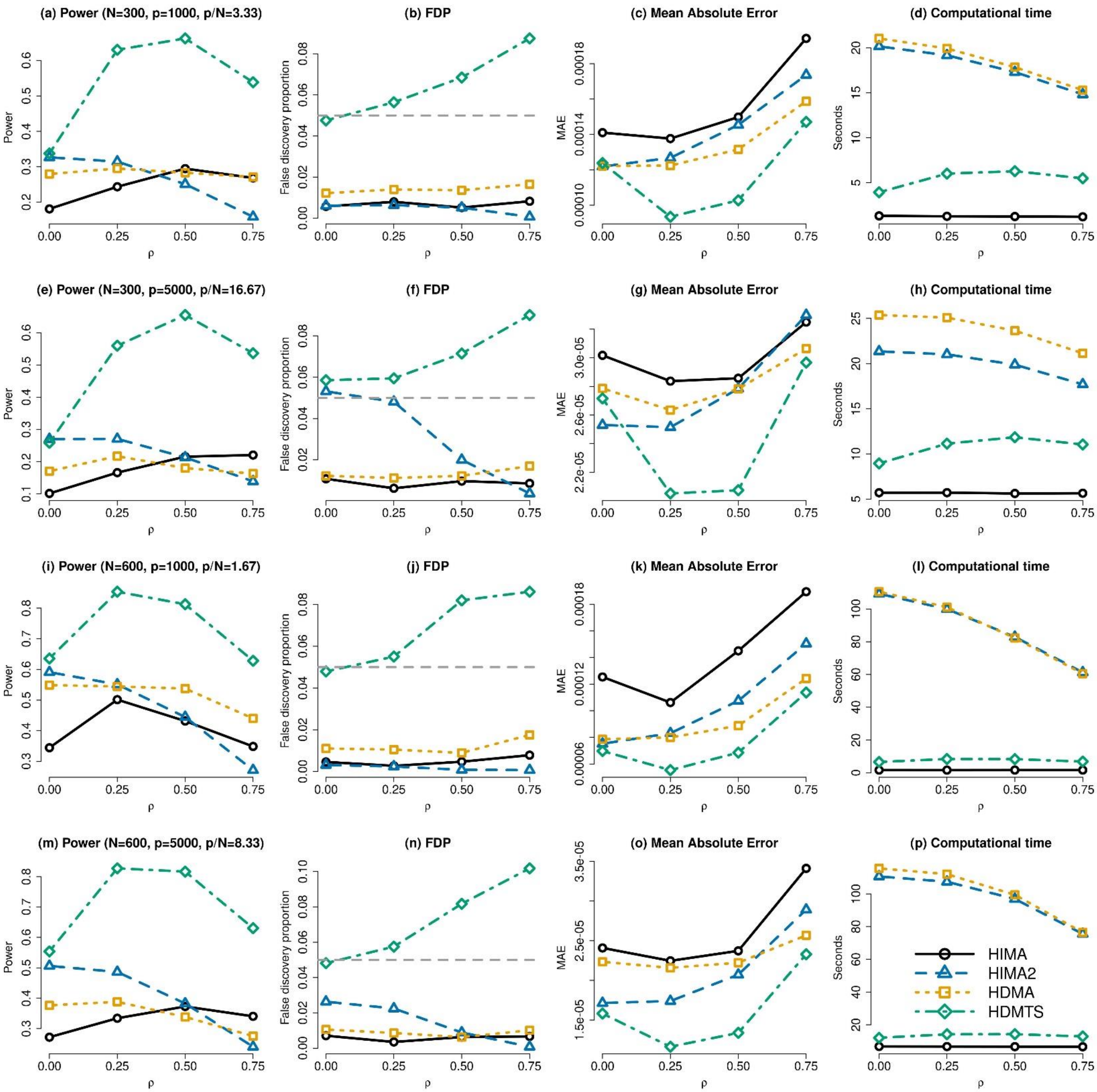

Figure S9. **Block-structured correlation scenario: Simulation results under the simulation setting using half of the $\boldsymbol{\beta_j}$ values (*j* = 1, …, *p*)**

Results are averaged over 1,000 simulation replications. The columns (from left to right) show statistical power, false discovery proportion (FDP), mean absolute error (MAE) of the estimated mediation effect, and computational time (seconds). The rows (from top to bottom) correspond to the settings: $N$=300, $p$=1000; $N$=300, $p$=5000; $N$=600, $p$=1000; and $N$=600, $p$=5000. The $x$-axis denotes correlation levels among CpG sites ($\rho = 0, 0.25, 0.50, or\ 0.75$). The gray horizontal line in the FDP panels indicates the nominal FDR level of 0.05.

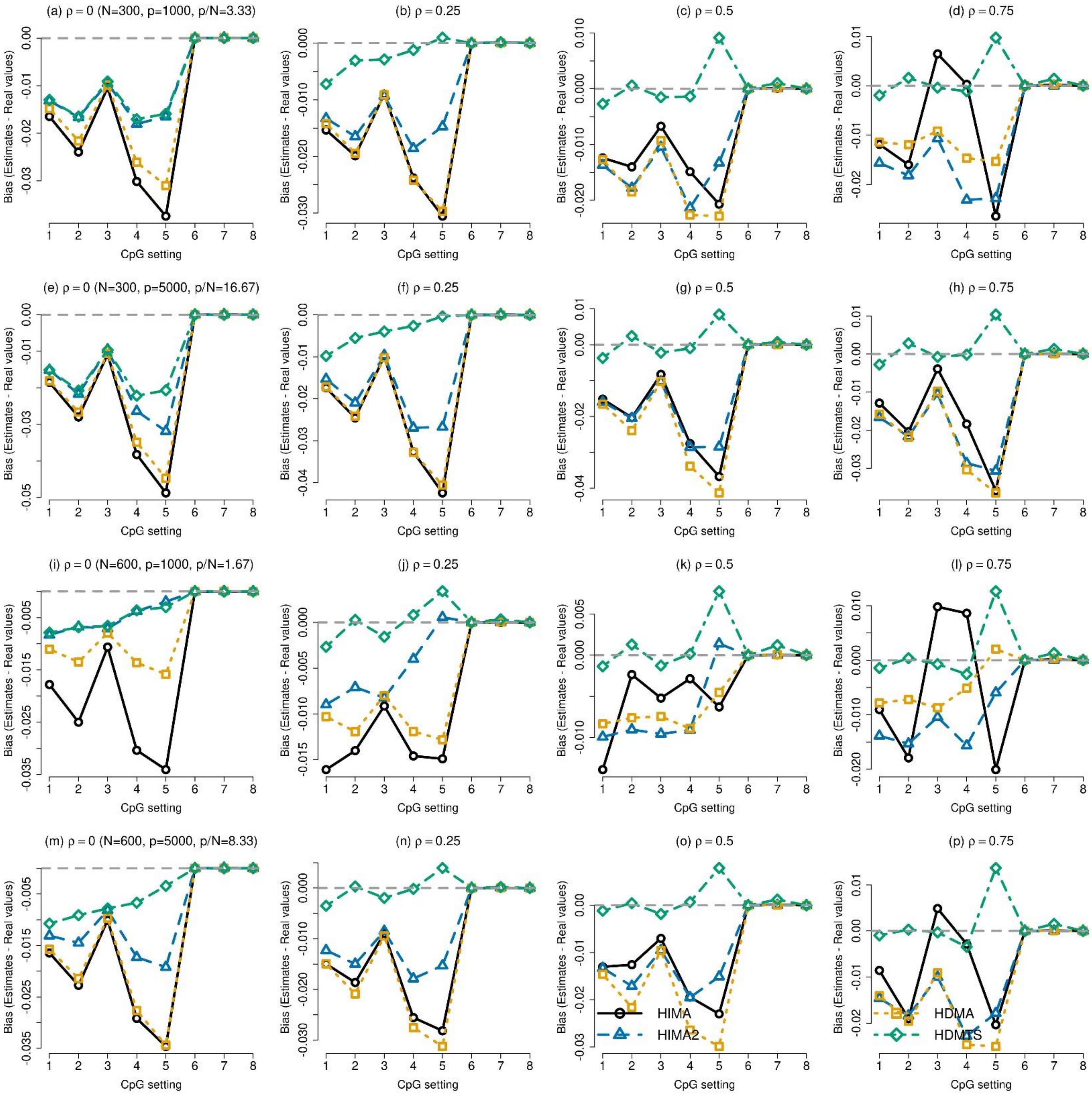


Figure S10. **Block-structured correlation scenario: Bias of estimated mediation effects under the simulation setting using half of the $\boldsymbol{\beta_j}$ values (*j* = 1, …, *p*)**

The rows (from top to bottom) correspond to the settings: *N*=300, *p*=1000; *N*=300, *p*=5000; *N*=600, *p*=1000; and *N*=600, *p*=5000. The columns (from left to right) represent correlation levels among CpG sites ($\rho = 0, 0.25, 0.50, or\ 0.75$). Bias was calculated as the average difference between estimated and true mediation

effects over 1,000 simulation replications. The *x*-axis denotes CpG settings corresponding to true nonzero effects: $\alpha_1 = 0.20$, $\alpha_2 = 0.25$, $\alpha_3 = 0.15$, $\alpha_4 = 0.30$, $\alpha_5 = 0.35$, $\alpha_7 = 0.10$, and $\alpha_j = 0$ otherwise; $\beta_1 = 0.10$, $\beta_2 = 0.125$, $\beta_3 = 0.075$, $\beta_4 = 0.15$, $\beta_5 = 0.175$, $\beta_6 = 0.05$, and $\beta_j = 0$ otherwise. CpG setting 8 represents the average bias across all CpG sites with $j \geq 8$. The gray horizontal lines indicate zero bias.

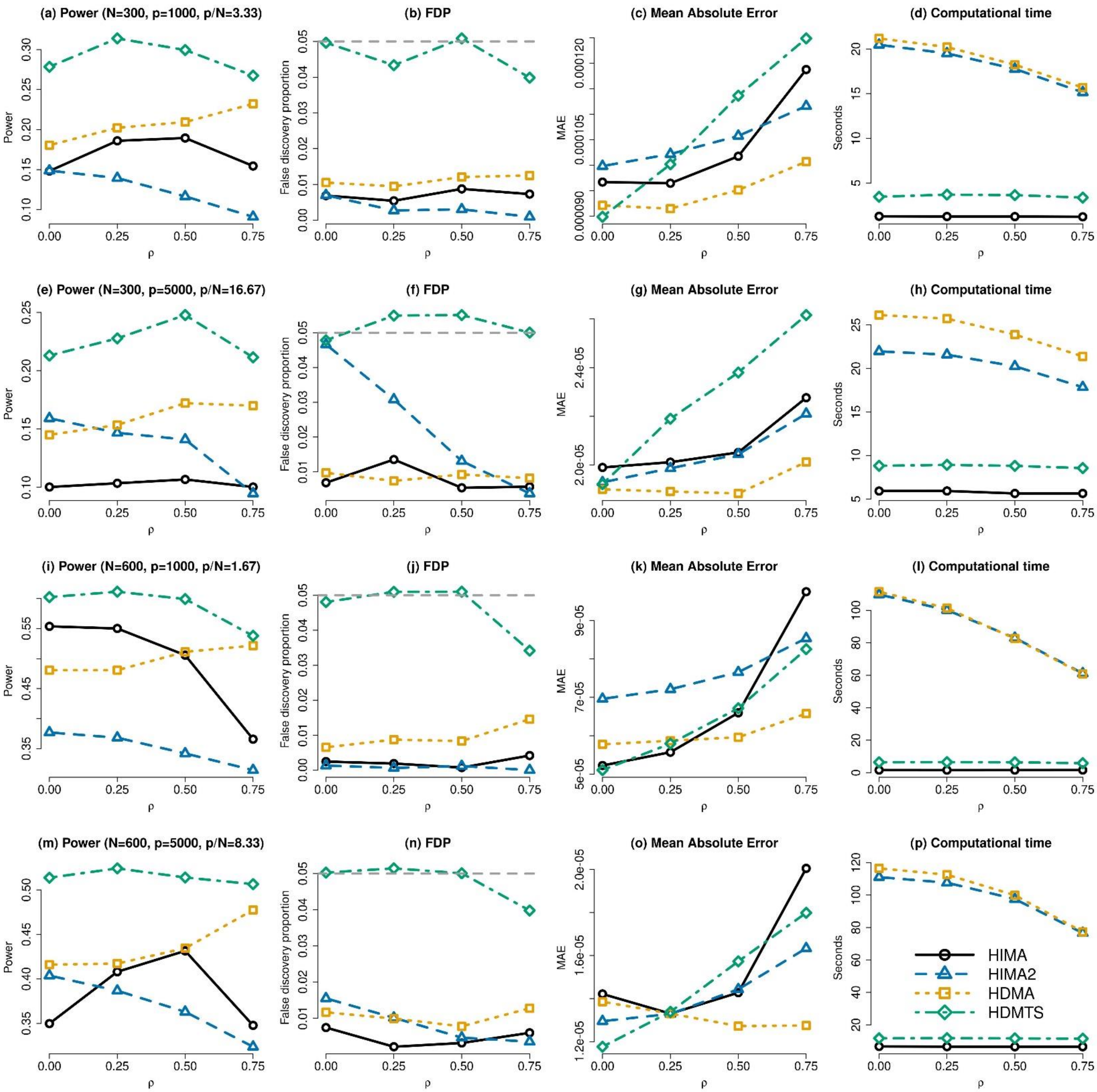

Figure S11. **Block-structured correlation scenario: Simulation results under the simulation setting using one-third of the $\alpha_j$ values (*j* = 1, …, *p*)**

Results are averaged over 1,000 simulation replications. The columns (from left to right) show statistical power, false discovery proportion (FDP), mean absolute error (MAE) of the estimated mediation effect, and computational time (seconds). The rows (from top to bottom) correspond to the settings: *N*=300, *p*=1000; *N*=300, *p*=5000; *N*=600, *p*=1000; and *N*=600, *p*=5000. The *x*-axis denotes correlation levels among CpG sites ($\rho = 0, 0.25, 0.50, or\ 0.75$). The gray horizontal line in the FDP panels indicates the nominal FDR level of 0.05.

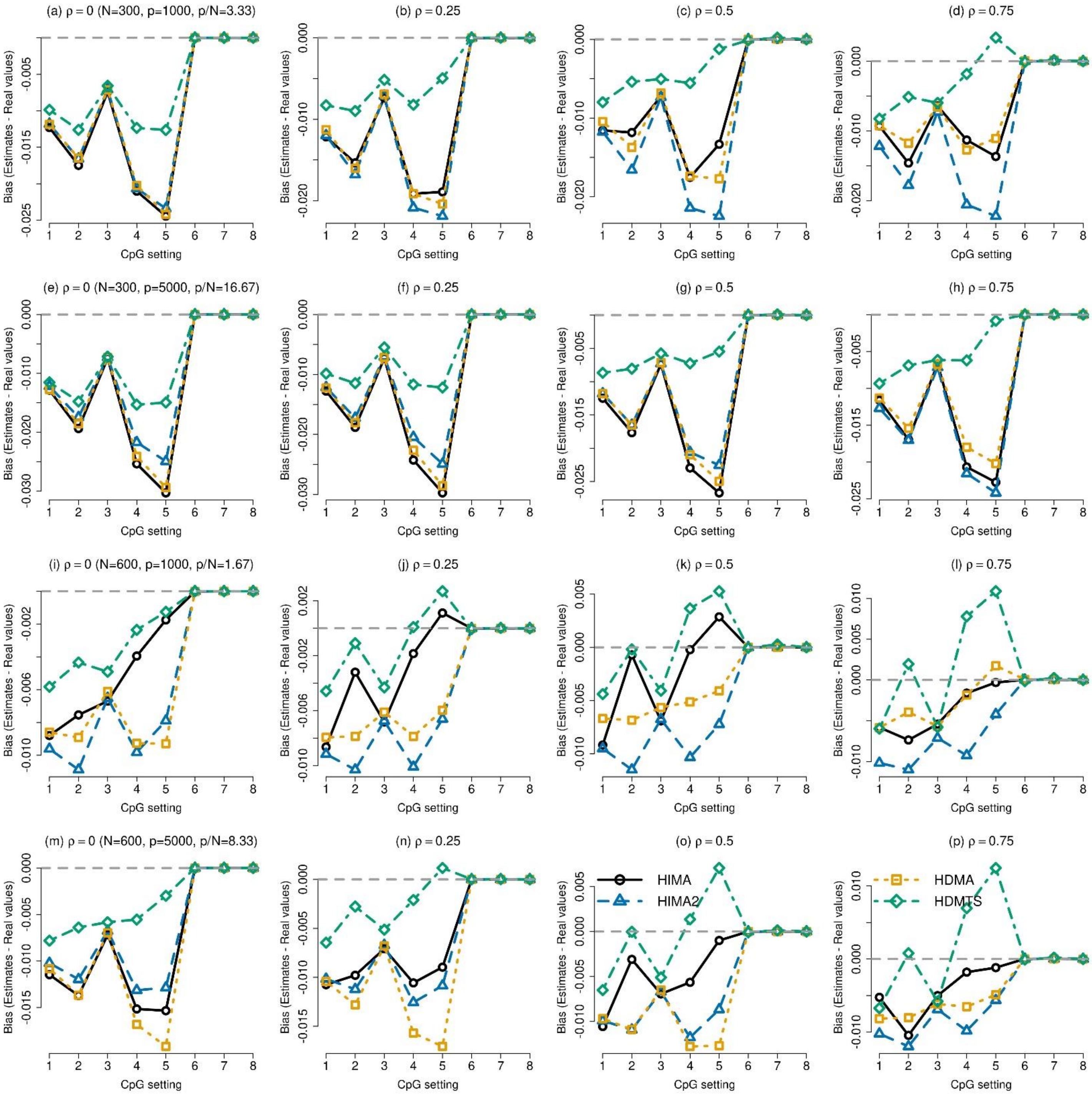


Figure S12. **Block-structured correlation scenario: Bias of estimated mediation effects under the simulation setting using one-third of the $\alpha_j$ values ($j$ = 1, …, $p$)**

The rows (from top to bottom) correspond to the settings: *N*=300, *p*=1000; *N*=300, *p*=5000; *N*=600, *p*=1000; and *N*=600, *p*=5000. The columns (from left to right) represent correlation levels among CpG sites ($\rho = 0, 0.25, 0.50, or\ 0.75$). Bias was calculated as the average difference between estimated and true mediation

effects over 1,000 simulation replications. The *x*-axis denotes CpG settings corresponding to true nonzero effects: $\alpha_1 = 0.067$, $\alpha_2 = 0.083$, $\alpha_3 = 0.05$, $\alpha_4 = 0.10$, $\alpha_5 = 0.117$, $\alpha_7 = 0.033$, and $\alpha_j = 0$ otherwise; $\beta_1 = 0.20$, $\beta_2 = 0.25$, $\beta_3 = 0.15$, $\beta_4 = 0.30$, $\beta_5 = 0.35$, $\beta_6 = 0.10$, and $\beta_j = 0$ otherwise. CpG setting 8 represents the average bias across all CpG sites with $j \geq 8$. The gray horizontal lines indicate zero bias.

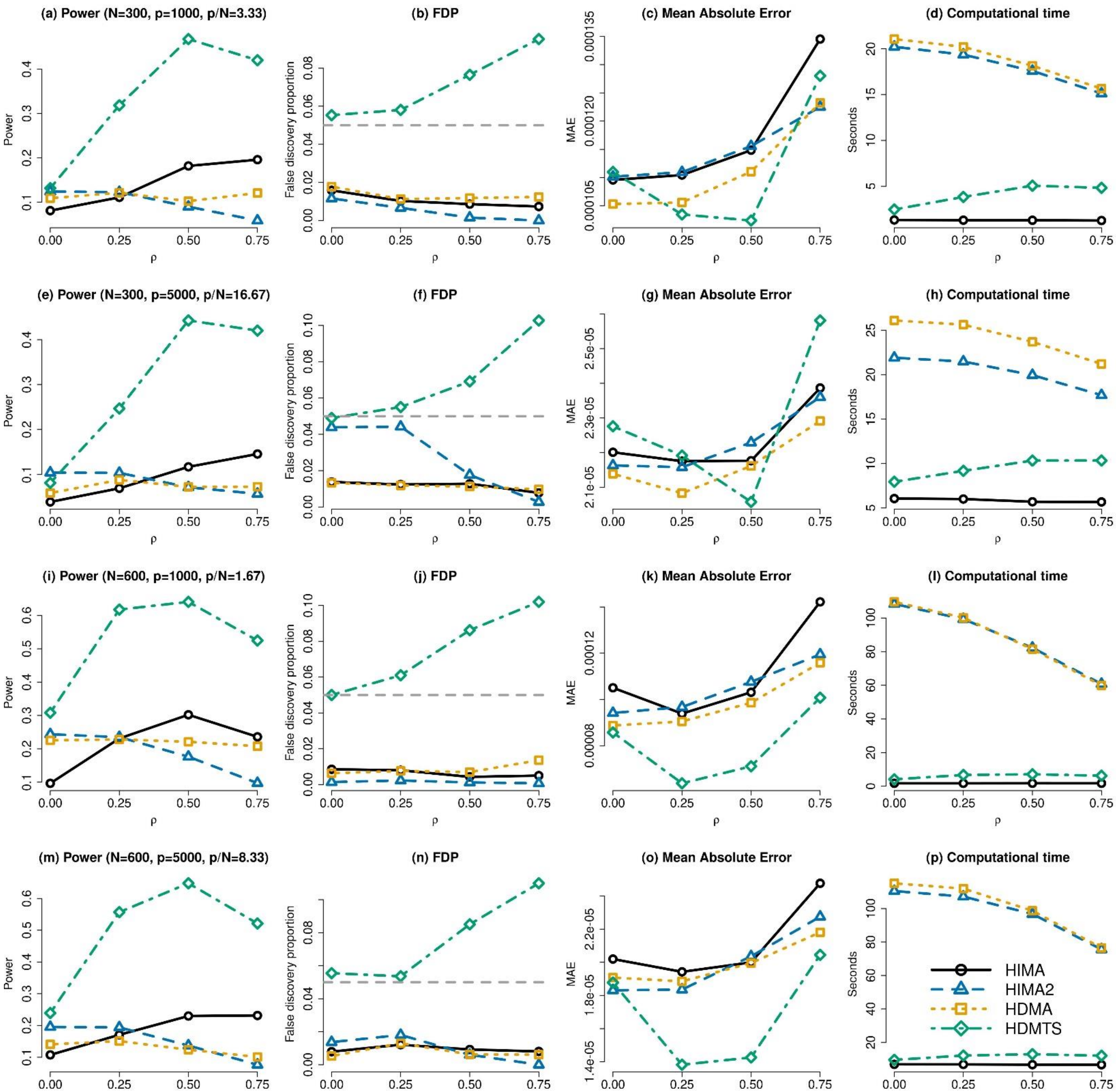

Figure S13. **Simulation results under the simulation setting using one-third of the $\boldsymbol{\beta_j}$ values (*j* = 1, …, *p*)**

Results are averaged over 1,000 simulation replications. The columns (from left to right) show statistical power, false discovery proportion (FDP), mean absolute error (MAE) of the estimated mediation effect, and computational time (seconds). The rows (from top to bottom) correspond to the settings: *N*=300, *p*=1000; *N*=300, *p*=5000; *N*=600, *p*=1000; and *N*=600, *p*=5000. The *x*-axis denotes correlation levels among CpG sites ($\rho = 0, 0.25, 0.50, or\ 0.75$). The gray horizontal line in the FDP panels indicates the nominal FDR level of 0.05.

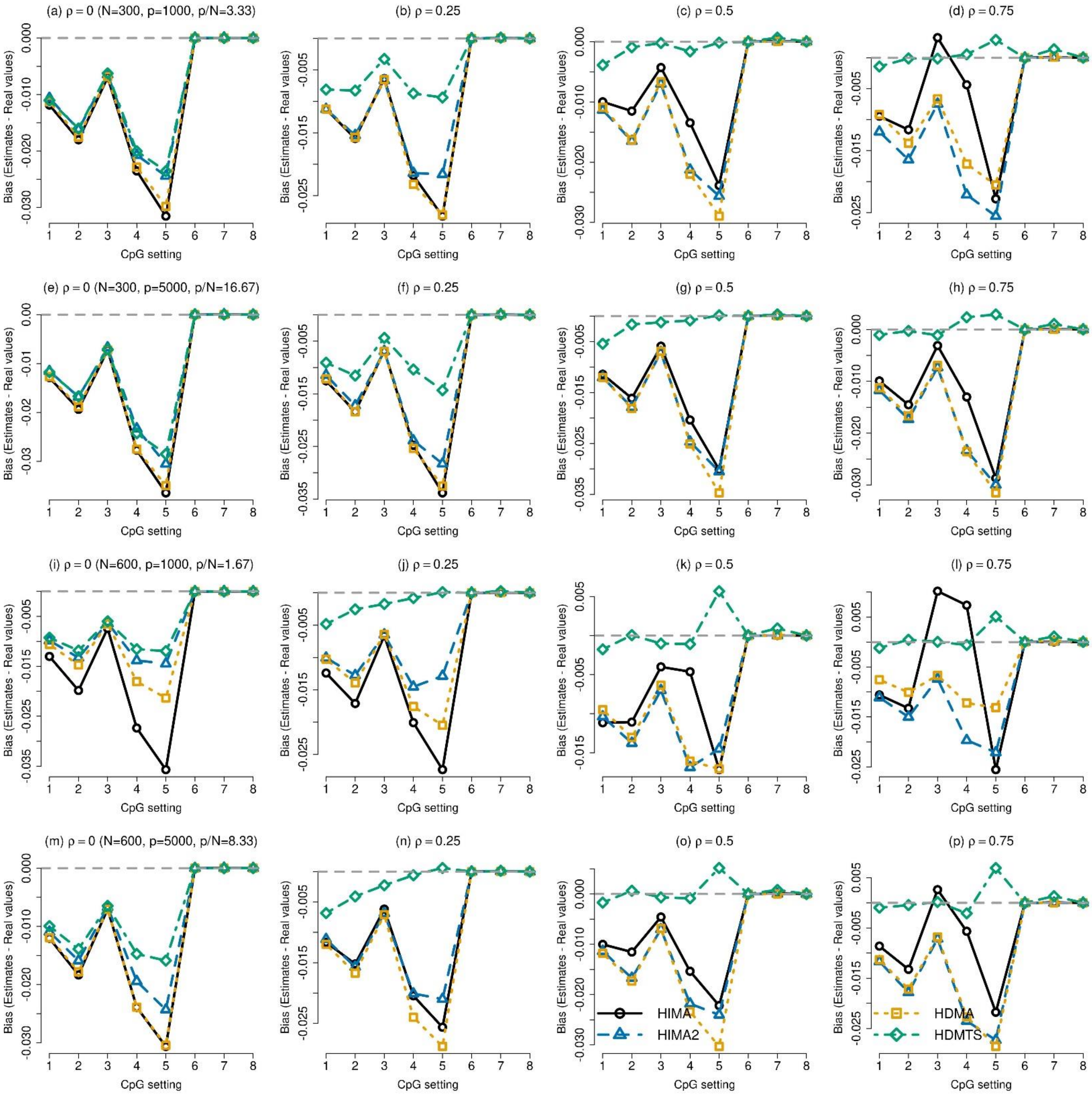


Figure S14. **Block-structured correlation scenario: Bias of estimated mediation effects under the simulation setting using one-third of the $\beta_j$ values (*j* = 1, …, *p*)**

The rows (from top to bottom) correspond to the settings: *N*=300, *p*=1000; *N*=300, *p*=5000; *N*=600, *p*=1000; and *N*=600, *p*=5000. The columns (from left to right) represent correlation levels among CpG sites ($\rho = 0, 0.25, 0.50, or\ 0.75$). Bias was calculated as the average difference between estimated and true mediation

effects over 1,000 simulation replications. The *x*-axis denotes CpG settings corresponding to true nonzero effects: $\alpha_1 = 0.20$, $\alpha_2 = 0.25$, $\alpha_3 = 0.15$, $\alpha_4 = 0.30$, $\alpha_5 = 0.35$, $\alpha_7 = 0.10$, and $\alpha_j = 0$ otherwise; $\beta_1 = 0.067$, $\beta_2 = 0.083$, $\beta_3 = 0.05$, $\beta_4 = 0.10$, $\beta_5 = 0.117$, $\beta_6 = 0.033$, and $\beta_j = 0$ otherwise. CpG setting 8 represents the average bias across all CpG sites with $j \geq 8$. The gray horizontal lines indicate zero bias.
